# An Emerging Class of Long Non-coding RNA With Oncogenic Role Arises From the snoRNA Host Genes

**DOI:** 10.3389/fonc.2020.00389

**Published:** 2020-04-07

**Authors:** Alina-Andreea Zimta, Adrian Bogdan Tigu, Cornelia Braicu, Cristina Stefan, Calin Ionescu, Ioana Berindan-Neagoe

**Affiliations:** ^1^Medfuture Research Center for Advanced Medicine, Iuliu Hatieganu University of Medicine and Pharmacy, Cluj-Napoca, Romania; ^2^Research Center for Functional Genomics, Biomedicine and Translational Medicine, Iuliu Hatieganu University of Medicine and Pharmacy, Cluj-Napoca, Romania; ^3^African Organisation for Research and Training in Cancer, Cape Town, South Africa; ^4^Surgical Department, Municipal Hospital, Cluj-Napoca, Romania; ^5^Department of Surgery, Iuliu Hatieganu University of Medicine and Pharmacy, Cluj-Napoca, Romania; ^6^Department of Functional Genomics and Experimental Pathology, The Oncology Institute “Prof. Dr. I. Chiricuta”, Cluj-Napoca, Romania

**Keywords:** lncRNAs, SNHG, snoRNA, cancer, malignant disease, proliferation, invasion

## Abstract

The small nucleolar RNA host genes (SNHGs) are a group of long non-coding RNAs, which are reported in many studies as being overexpressed in various cancers. With very few exceptions, the SNHGs (SNHG1, SNHG3, SNHG5, SNHG6, SNHG7, SNHG12, SNHG15, SNHG16, SNHG20) are recognized as inducing increased proliferation, cell cycle progression, invasion, and metastasis of cancer cells, which makes this class of transcripts a viable biomarker for cancer development and aggressiveness. Through our literature research, we also found that silencing of SNHGs through small interfering RNAs or short hairpin RNAs is very effective in both *in vitro* and *in vivo* experiments by lowering the aggressiveness of solid cancers. The knockdown of SNHG as a new cancer therapeutic option should be investigated more in the future.

## Introduction

Worldwide, in 91 developed countries, cancer is the first or second leading cause of mortality with 18.1 million new cases and 9.5 million deaths estimated in 2018 (1). According to Siegel et al., in the United States, between 2000 and 2014, the most frequently diagnosed types of cancer in men are: prostate, lung, and colorectal cancer; while in women, these are: breast, lung, and colorectal cancer (2). These statistics show that the malignant disease has a high impact over the socioeconomical welfare of developed countries; thus, each new discovery of its molecular context should be carefully analyzed and evaluated for its potential as biomarker and/or therapeutic target.

In malignant diseases, the imbalance between overactivation of proto-oncogenes and inhibition of tumor suppressor genes is usually the consequence of a faulty regulatory system, which includes, among others, the non-coding RNAs (3–5). Most of these transcripts do not have the ability to interact with the ribosome and are not translated into proteins (6–11); however, they interact with other coding or non-coding RNAs (ncRNAs), being part of complex regulatory networks (12). The ncRNAs are divided into two classes: the small ncRNAs (with <200 nt) and the long ncRNAs (lncRNAs), with >200 nt (13). The short ncRNAs are generated through multistep processing of the primary transcript, while the lncRNAs remain highly similar to their primary transcripts (14–17). The short ncRNAs are divided into: small interfering RNAs (siRNAs), microRNAs (miRNA), piwi-interacting RNAs (piRNAs), and others (18). The lncRNAs, even if very heterogeneous in terms of biogenesis, structure, mechanism of action, and biological functions, are still mentioned under the general term of lncRNAs (6, 19, 20).

The snoRNAs (small nucleolar RNAs) were discovered in the late 1980s (21). However, only recently these transcripts were considered as potential biomarkers of cancer (22). The snoRNAs have the general characteristics of small ncRNAs, with the processed transcripts having between 65 and 300 nt. The snoRNAs do not have a poly-A tail and are 5′Cap, which means that they are not exported from the nucleus. They are transcribed from clusters of protein-coding genes or genes coding for other ncRNAs, and a small franction of snoRNAs are originated in the intergenetic regions. Only three snoRNAs (U3, U8, and U13] and telomerase snoRNA are transcribed and processed as single units. The polycistronic transcription of snoRNAs has a phylogenetic progression, which means that in higher organisms, more snoRNAs originating from clusters of genes are found (23).

There are two types of snoRNAs: C/D box snoRNA and H/ACA snoRNA. The C/D box snoRNAs have a C box (RUGAUGA, R = A/G) and two D boxes (CUGA). The H/ACA snoRNAs contain the H box (ANANNA, N = A/T/C/G) and the ACA sequence (24). The C/D box snoRNAs are involved in the 2-O′-methylation of rRNA, and the H/ACA snoRNA cause the pseudouridylation of rRNAs. Some snoRNAs are further processed into microRNAs, which repress the translation of mRNA in the cytoplsm (23) or into piwi-interacting RNAs, essential in the regulation of transposomes mobility in the nucleus (25).

The snoRNA host genes are mainly located in the introns of protein-coding genes, with ancestral positionally conserved (APC) snoRNA sequences constituting only 2% of host genes (26). However, the intronic sequences of snoRNAs show a high degree of conservation across species (27) in comparioson with intergenetic regions (26). The generation of snoRNAs across species may be a result of mobility of the nested snoRNAs in protein-coding genes, since orthologous introns can encode for non-orthologous snoRNAs in different species. However, as the new element of non-coding sequence moves in a protein-coding gene, it results in loss-of-function genetic variants (28).

Some snoRNA genes, without any capacity to code for proteins, still contain both introns and exons in their sequences However, snoRNAs are generated only from introns. If the full-length transcript, including exons, is kept, it will function as a type of lncRNA, named small nucleolar RNA host gene (SNHG) (29). A pan-cancer analysis of 31 cancer types based on “omics” data retrieved from TCGA revealed that the snoRNA host genes are enriched in non-protein coding genomic regions and that these have higher expression (30). There seems to be a positive correlation between SNHGs and their corresponding snoRNAs. More precisely, the intracellular level of snoRNAs is influenced by their structure and the availability of SNHGs (23). In murine genome, *Zfas1* is an antisense transcript, which shares a bidirectional promoter with the protein coding gene Znfx1 (zinc finger NFX-1-type containing), their expression in different tissues being positively correlated. Zfas is also an lncRNA from an snoRNA gene, and it can be processed into three types of intronic snoRNAs: SNORD12, SNORD12b, and SNORD12c. SNORD12b has a higher expression than SNORD12 and SNORD12c, in cells with a lower degree of differentiation, due to a higher stability of the processed structure, which contains an additional short hairpin structure. The snoRNAs are located only in the nucleus, whereas Zfas is present in the cytoplasm and nucleus as well. The siRNA knockdown of Zfas only slightly affected the expression of snoRNAs (31). In addition, SNHG12 knockdown in hepatocellular carcinoma did not statistically significantly affect the expression of its snoRNAs (SNORA44, SNORA61, SNORA16A, and SNORD99] (32). However, the expression of some SNHGs, such as SNHG17, is regulated by the copy number variations (CNV) of their host gene, this being also correlated with the aggressiveness of non-small cell lung cancer and squamous cell lung cancer. A change in SNHG intracellular level can generate a significant downstream effect, such as siRNA inhibition of SNHG17 that caused 637 protein coding RNAs to be up-regulated and 581 to be down-regulated (33).

The proteins that target the SNHGs can also target their corresponding snoRNAs. A recent article reported that p53 activation repressed the intracellular level of SNHG1 that also reduces the level of snoRNAs: SNORD22, SNORD25, SNORD26, SNORD27, SNORD28, and SNORD75. The SNORD28 is further processed into an miRNA, named sno-miR-28. This miRNA repressed the expression of TAF9B, a coactivator of p53. As follows, a positive feedback loop forms between sno-miR-28-p53-SNHG1-SNRD28 (34).

**Figure 1:**
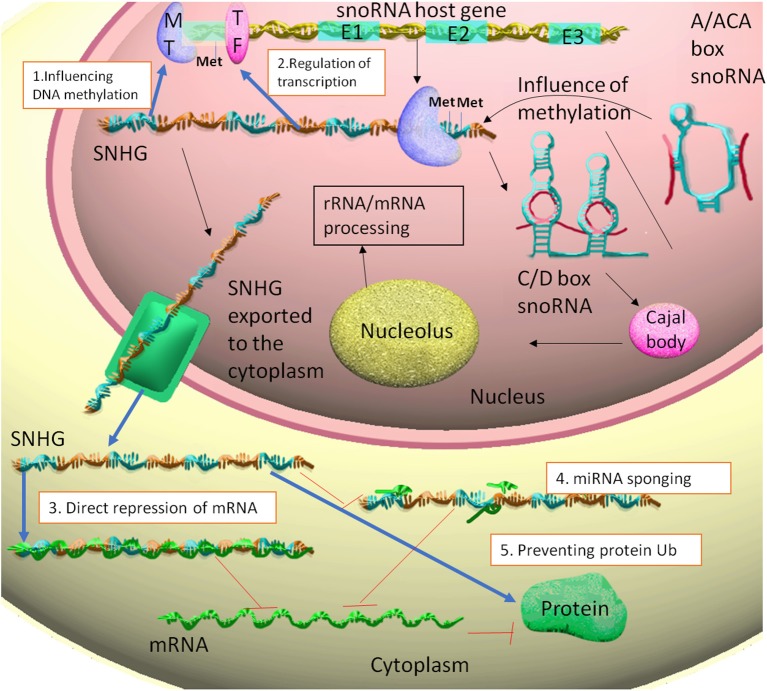
The snoRNA host gene are transcribed to SNHG. Some of these transcripts generate from their introns C/D box snoRNAs or A/ACA box snoRNAs. The snoRNAs can influence the methylation of their SNHG of origin. The SNHGs are then associated with Cajal bodies and nucleolus, thus participating in rRNA and mRNA primary processing. The SNHGs in the nucleus fulfill the following functions: 1. Influencing DNA methylation through interaction with methyltransferases (MT), such as EZH2. 2. Regulating transcription through interaction with transcription factors (TF), such as E2F1. The SNHGs are then exported into the cytoplasm, where: 3. Can directly interact and repress mRNA translation; 4. act as ceRNA for miRNAs (miRNA sponging) and indirectly up-regulating the translation of miRNA targets. 5. Stabilizing proteins, by preventing protein ubiquitination.

The cellular location differs between snoRNAs and SNHGs. While the snoRNAs remain in the nucleus (35), SNHGs are present in the nucleus as well as in the cytoplasm (29) (Figure 1).

Through our research of literature on SNHGs, we have identified five main types of molecular mechanism of action, with different cellular localizations.

Nuclear:

1. Influencing DNA methylation through modulation of methylation enzymes
2. Interaction with transcription factors and repression of gene transcription.

Cytoplasm:

3. MiRNA sponging and the releasing of miRNA targets
4. Direct binding to the mRNA and repression of translation
5. Prevention of protein ubiquitination through single protein interaction or multi-protein complex.

In the nucleus, SNHG1 binds to the Mediator complex and facilitates the interaction of the enhancer and promoter DNA region corresponding to *SLC3A2* gene (36). *SLC3A2* is a well-known cancer-promoting gene (37, 38). The Mediator complex is composed of multiple proteins and non-coding RNAs that cooperate with RNA polymerase II during transcription initiation (39). SNHG1 competitively binds to the Far Upstream Element Binding Protein 1 (FUBP1), and it prevents FUBP1 interaction with its repressor, followed by overstimulated transcription of c-MYC gene (39).

In hepatocellular carcinoma, SNHG6 lowers DNA methylation through inhibition of S-adenosylmethionine and down-regulation of MAT1A (methionine adenosyltransferase 1A). SNHG3 binds to enhancer of zeste homolog 2 (EZH2) and causes the methylation of several genes (40, 41).

In the cytoplasm, SNHGs fulfill the role of competing endogenous RNAs (ceRNAs) for miRNAs (42). ceRNA is a term used for the non-coding RNA species that share common miRNA binding sites with mRNAs. The process is also known under the name of miRNA sponging (43). The SNHGs can also interact directly to the mRNA and cause their overexpression, such as in the case of SNHG1 and p53 in colorectal cancer (44).

The SNHGs can also post-translationally interact with proteins and prevent their ubiquitination. This is the case of SNHG15 and SLUG in colorectal cancer (45).

The SNHGs have an oncogenic role in cancer through the versatility of their interactions at DNA–RNA–protein level. The correlation between SNHGs and cancer still lacks important information on the general activity of SNHGs, mainly because the data are still fractioned and focused on each SNHG. In current article, we reviewed over 200 articles on the role of SNHG in different malignancies. We first briefly consider the oncogenic activity of each SNHG separately, and in the final section of the article, we draw some general lines of consideration on the realistic potential of SNHGs to reach clinical applications, in the context of a comprehensive overview of their biological influence. To our knowledge, this is the first time that a comprehensive clarification of the general mechanism of action of SNHGs in cancer is given.

## Small Nucleolar RNA Host Gene 1 (SNHG1)

SNHG1 generates, through alternative splicing, eight snoRNAs: SNORD22 (46), SNORD25, SNORD26, SNORD27, SNORD28, SNORD29, SNORD30, and SNORD31 (47).

In colorectal cancer, SNHG1 leads to poor prognosis (44, 48, 49). It promotes cancer cell viability, proliferation, cell cycle progression, tumor growth, and increased invasion/migration capacity (44, 48–50). Its mechanism of action consists of targeting the p53 gene (44) and overstimulating the Wnt/β-catenin signaling pathway (48, 50]. It also acts as a ceRNA for miR-145 (50), a suppressor of a colorectal cancer invasion and migration (51).

In ovarian cancer, SNHG1 promotes cell proliferation, invasion, and new colony formation, while impairing cell apoptosis (52). In prostate cancer, SNHG1 acts as an miR-199a sponge, thus increasing the level of CDK7 (cyclin-dependent kinase 7), which stimulates cell division (53), and HIF-1α (hypoxia-inducible factor 1-alpha), which leads to enhanced angiogenesis (54). HIF-1α is a transcription factor (TF) activated in case of hypoxic microenvironment present at the tumor site. This TF stimulates the expression of VEGFα, an important promoter of tumor neoangiogenesis. This process is essential for tumor survival (provision of nutrients) and spreading to new sites of the body (connection with systemic blood circulation) (55).

In esophageal cancer, SNGH1 sponges miR-338 (42) miR-338 is involved in stimulating the radiotherapy-induced apoptosis of esophageal cancer cells, by targeting *Survivin* gene (56). SNHG1 in esophageal cancer promotes cell proliferation and EMT (epithelial to mesenchymal transition)-mediated invasion (56), by down-regulation E-CAD (E-cadherin) and up-regulation VIM (Vimentin) and N-CAD, as well as activation of Notch signaling pathway (57). EMT is the process through which epithelial cells lose their tight junctions and cell polarity, while acquiring a higher degree of mobility and ability of invasion through basement membrane. There are three types of EMTs: (1) during embryogenesis, this process is necessary for the transformation of epiblast into mesinchyme; (2) during wound healing and chronic inflammation when an additional supply of fibroblasts are locally needed; (3) during cancer progression, when the cells from the *in situ* primary tumor invade the local lymph nodes or enter into systemic blood circulation in order to from distant metastasis (58). E-cadherin is a cell adhesion molecule (CAM) specific for the formation of adherent junctions between epithelial cells. It is also considered as a tumor suppressor through DNA methylation of oncogenes (59). N-CAD suppresses the activity of E-CAD and stimulates the motility of cells. During embryonic development, it supports gastrulation and the formation of neuronal crest; however, N-CAD can be reactivated during malignant development to support EMT progression (60). VIM is a component of the intermediate filaments from cytoskeleton that keep the organelles in place, while also allowing to some degree their mobility. VIM is a highly flexible component found in all cells, but its overexpression is specific for mesenchymal cells. In cancer, VIM is a major stimulator of EMT and inhibitor of autophagy through its repression of Beclin 1 and 14-3-3 (61).

In hepatocellular carcinoma, SNHG1 was associated with advanced stages, larger tumor size, and poor differentiation due to its interaction with the tumor suppressor p53 (62). SNHG1 sponges miR-195 (63), a miRNA that inhibits metastasis and angiogenesis in hepatocellular carcinoma by targeting *FGF2* (fibroblast growth factor) and *VEGFA* (vascular endothelial growth factor alpha) (62, 64). Similarly, SNHG1 overexpression results in worse prognosis in glioma because it increases proliferation and invasion while inhibiting apoptosis (65).

In lung cancer, SNHG1 acts as a tumor-promoter by sponging miR-145 (66) and miR-101 (67) and enhancing the Wnt/β-catenin signaling pathway (67). miR-145 acts as a tumor suppressor in lung cancer by impairing cell migration and invasion (68, 69). In lung cancer, miR-101 underexpression is associated with advanced stages and lymph node metastasis and it induces cell apoptosis by targeting *MCL-1* (70).

In osteosarcoma, SNHG1 promotes cell proliferation, tumor growth, invasion, and EMT by sponging miR-326, resulting in overexpression of *NOB1* [NIN1 (RPN12) Binding Protein 1 Homolog] (70). miR-326 suppresses the antiapoptotic gene BCL-2 in osteosarcoma and acts as a tumor suppressor (71). BCL-2 is a major antiapoptotic marker that inhibits the liberation of cytochrome C from mitochondria (72), thus impairing the cleavage of the effector caspases CASP3 and CASP7.

SNHG1 also represses miR-577 and activates WNT/β-catenin pathway (73). WNT2B, a member of WNT signaling pathway, plays a significant oncogenic role in osteosarcoma (73). Further research and details regarding the latest studies on SNHG1 oncogenic function in malignant disease are presented in Supplementary Table 1.

## Small Nucleolar RNA Host Gene 3 (SNHG3)

SNHG3 is an oncogenic lncRNA that generates SNORD17 (74).

In osteosarcoma, SNHG3 overexpression facilitates cell invasion and migration *in vitro*, through the inhibition of miR-151a-3p (75) and miR-196a-5p (76).

In glioma, SNHG3 induces a more aggressive phenotype through increased cell proliferation and apoptosis resistance of malignant cells. SNHG3 interacts with the protein EZH2, which determines the methylation of KLF2 (Krüppel-like Factor 2), and p21 gene promoters, which inhibit their transcription through an epigenetic mechanism (40). KLF2 is a zinc-finger transcription factor that activates CD4+ T cells (77). As a tumor suppressor, KLF2 also impairs invasion and stimulates apoptosis through down-regulation of MMP9 and BCL-2 (78). P21 is a major tumor suppressor activated by p53 in cancer. During cell cycle, p21 activates CDK4 (cyclin-dependent kinase 4) and CDK6 (cyclin-dependent kinase 6), resulting in transition of cells from G1 to S phase. During S phase, however, it impairs the interaction between CDK2 (cyclin-dependent kinase 2) and cyclin E, hence stopping the transition of cells from S to G2 phase. Moreover, in G2, P21 also functions as a disruptor of CDK1 (cyclin- dependent kinase 1) and Cyclin B1 interaction, thus preventing mitosis initiation (79). In gastric cancer, SNHG3 binds to EZH2 and epigenetically silence MED18 (Mediator Complex Subunit 18) expression (41).

Furthermore, SNHG3 causes proliferation of colorectal cancer cells by sponging miR-182 and allowing the overexpression of the tumor promoting transcription factor c-MYC (80). In hepatocellular carcinoma, SNHG3 is positively associated with larger tumor size and relapse (81), in addition to Sorafenib resistance, as a consequence of ceRNA activity on miR-128. Further research and details on the role of SNHG3 in malignant diseases are presented in Supplementary Table 2.

## Small Nucleolar RNA Host Gene 5 (SNHG5)

SNHG5 is processed into SNORD 50 and SNORD50′ (82).

In SNHG5, overexpression increases the ability of breast cancer cells to proliferate and go through cell cycle, by releasing PCNA (proliferating cell nuclear antigen) from the inhibition of miR-154-5p (83).

In bladder cancer, SNHG5 induces p27 silencing, followed by enhanced proliferation rate and cell cycle progression, associated with apoptosis inhibition (84). In chronic myelogenous leukemia (CML), SNHG5 up-regulation stimulates imatinib resistance, through down-regulation of miR-205 and up-regulation of *ABCC2* (ATP binding cassette subfamily C member 2) (85). In CML patients with rare BCR-ABL variants, this microRNA was found to be down-regulated in imatinib resistant patients compared with imatinib responsive patients (86).

In gastric cancer, there are controversial results. One study reported SNHG5 as an oncogene that stimulates cell proliferation and migration, by sponging miR-32 and up-regulation of KLF4 (Krüppel-like factor 4) (87), while another study reported that SNHG5 is a tumor suppressor, which interacts with MTA2 (metastasis associated 1 family member 2), and it impairs the MTA2 translocation from the cytoplasm to the nucleus. The overexpression of SNHG5 was also associated with a lower level of MMP9 (metalloproteinase 9), MMP1 (metalloproteinase 1), MMP13 (metalloproteinase 13), and EGFR (epithelial growth factor receptor) (88). MTA2 is a major repressor of E-CAD; thus, it stimulates EMT (89). MMPs are a class of metalloproteinases that degrade non-cellular components of extracellular matrix, such as collagen, gelatin, lamins, and others. The secretion of MMPs helps malignant cells to degrade the basement membrane from their site of origin and to get access to the local blood vessels and lymphatic system (90).

In colorectal cancer, SNHG5 interacts with the SPATS2 (spermatogenesis associated serine rich 2) and increases its stability by blocking the mRNA degradation in the cytoplasm, caused by STATS2 mRNA interaction with STAU1 (staufen double-stranded RNA binding protein 1) (82).

Through miR-26 sponging and up-regulation of TRPC3 (transient receptor potential channel 3), SNHG5 stimulates melanoma cell growth. The exogenous knockdown of SNHG5 in melanoma cells *in vitro* decreases their proliferation rate and invasive capacity, while it stimulated apoptosis (91). Further research and details on the role of SNHG5 in malignant diseases are presented in Supplementary Table 3.

## Small Nucleolar RNA Host Gene 6 (SNHG6)

SNHG6 is the lncRNA of origin for U87 SNORD (92) and U88 small Cajal bodies (93).

SNHG6 has an oncogenic role in gastric cancer by promoting cell growth, migration, and EMT-mediated invasion. These effects are a consequence of miR-101 (94) and miR-26a sponging (95). In colorectal cancer, the shRNA inhibition of SNHG6 causes increased level of miR-181a-5p and decreased level of E2F5 (E2F transcription factor 5), a transcription factor, which further decreases the number of colonies and invasive cells, as well as cell cycle arrest during *in vitro* studies (96). SNHG6 sponging of miR-26a/b and miR-214 causes an increased level of EZH2 (enhancer of zeste homolog 2), which further affects the epigenetic landscape of the colorectal cells (97).

SNHG6 sponges miR-101 causing the EMT through up-regulation of N-CAD and VIM and down-regulation of E-CAD and β-catenin (94). By suppressing SOCS2 (suppressor of cytokine signaling 2), miR-101 inhibits *Helicobacter pylori*–induced gastric cancer tumorigenesis (98) and tumor growth (99).

SNHG6 level has a positive correlation with disease progression and formation of local lymph node metastasis in the case of esophageal carcinoma (100).

In hepatocellular carcinoma, SNHG6 promotes cell cycle progression and apoptosis evasion. miR-101 is targeted by SNHG6, which further stimulates ZEB1 (zinc finger E-box-binding homeobox 1) expression (101).

In osteosarcoma, SNHG6 exogenous suppression impairs cell autophagy, apoptosis, colony formation, and invasion through miR-26a-5p sponging, which results in ATF3 (activating transcription factor 3) up-regulation. ULK1 (autophagy activating kinase) is a positive up-stream regulator of SNHG6 (102). Further research and details on the role of SNHG6 in malignant diseases are presented in Supplementary Table 4.

## Small Nucleolar RNA Host Gene 7 (SNHG7)

SNHG7 is the origin for SNORA17 and SNORA43 (103).

SNHG7 is up-regulated in high grade bladder cancer. siRNA-mediated inhibition of SNHG7 leads to decreased wound closure speed in scratch assay, lower number of invasive cells in transwell assay, and an increased intracellular level of the proapoptotic marker BAX (Bcl-2-associated X protein), cell cycle inhibitor p21, and anti-invasive adhesion molecule, E-cadherin (104), while the expression of mesenchymal promoting proteins, namely, N-cadherin, VIM, and SNAIL are increased (105). SNHG7 activity in bladder cancer also extends to the activation of WNT/β-catenin pathway (106). BAX is an important proapoptotic marker. Its expression and activation are stimulated by p53 during apoptosis, when BAX is released from nucleus into the cytoplasm, where it binds to mitochondrial membrane. This is followed by mitochondrial pore formation necessary for the release of cytochrome C. BAX also directly interacts and represses the antiapoptotic protein BCL-2 (107). During WNT/β-catenin pathway activation, WNT protein binds to Frizzled receptor and stimulates its interaction with the LRP6 (lipoprotein receptor related protein 6). This is followed by accumulation and nuclear translocation of β-catenin. In the nucleus, β-catenin disrupts the interaction between T cell factor and lymphoid enhancer factor (TCF/LEF), thus stimulating the transcription of genes involved in cell self-renewal and proliferation capacity, necessary for tumorigenesis. In cancer, different components of this pathway acquire gain-of-function mutations (108).

In breast cancer, when comparing exogenous silencing of SNHG7 with controls, the following was observed: decreased self-renewal capacity of malignant cells, lower number of invasive cells during transwell assay, and smaller volume of xenograft tumors. At the molecular level, SNHG7 silencing lowered VIM and SNAIL level while increasing E-cadherin expression. SNHG7 overexpression leads to up-regulation of the antiapoptotic gene Survivin and the cell cycle promoting gene, Cyclin D, through the activation of Notch signaling pathway (109). In breast cancer, SNHG7 acts as ceRNA for several tumor suppressor miRNAs: miR-34a (109), miR-186 (110), and miR-381 (111).

Overexpression of SNHG7 stimulates the *in vitro* invasion capacity of colorectal cancer cell lines. The siRNA mediated silencing of SNHG7 increases cleaved PARP and cleaved Caspase 3 levels leading to apoptosis initiation. *In vivo*, colon cancer cells transfected with SNHG7 siRNA form smaller tumors, in comparison with negative control (112), while the overexpression of SNHG7 causes increased liver metastasis from primary colon tumors (113). In colon cancer, SNHG7 also targets miR-34a (112) and miR-216b (113).

In both esophageal (114) and gastric cancer, this lncRNA represses expression of p15 and p16, two tumor suppressors. Consequently, it stimulates proliferation and cell cycle progression and it inhibits apoptosis (114, 115).

In glioblastoma, SNHG7 induces proliferation, migration, and invasion of malignant cells, by inhibiting miR-5095 and by activating the WNT/β-catenin signaling pathway (116).

In hepatocellular carcinoma, inhibition of SNHG7 decreases the invasion capacity of malignant cells. The protein expression of RBM5 (RNA binding motif protein 5) was increased after SNHG7 silencing (117).

In melanoma, SNHG7 is overexpressed in malignant tissue vs. normal tissue. It increases cell invasion and migration capacity through positive correlation with SOX4 (SRY-box transcription factor 4) (118). miR-503, in prostate cancer, targets RNF31 and inhibits proliferation and metastasis (119). In prostate cancer, this lncRNA represses miR-503 and leads to tumor cell proliferation, cell cycle progression, and xenograft tumor growth (120).

In lung cancer, SNHG7 stimulates proliferation, migration, and invasion while inhibiting apoptosis through up-regulation of FAIM2 (Fas apoptotic inhibitory molecule 2) (121) and miR-193b sponging (122).

In neuroblastoma, it forms a positive feedback loop with miR-653-5p and STAT2 (signal transducer and activator of transcription 2), which sustains an aggressive phenotype of malignant cells (123). Further research and details on the role of SNHG7 in malignant diseases are presented in Supplementary Table 5.

## Small Nucleolar RNA Host Gene 12 (SNHG12)

From the introns of SNHG12, four different types of snoRNAs are processed: SNORA44, SNORA61, SNORA16A, and SNORD99 (32). Latest studies show the SNHG12 up-regulation, and the inhibition of tumor suppressor miRNAs, in many cancer types (Supplementary Table 6).

SNHG12 has various biological functions through targeting of cell proliferation, invasion, migration, and apoptosis. SNHG12 is involved in different types of malignant diseases by targeting the following microRNAs: miR-424-5p in cervical cancer (124), miR-320 in gastric cancer (125), miR-199a-5p in renal cell carcinoma (126), miR-199a/b in hepatocellular carcinoma (32), miR-199a/b in gastric cancer (127), miR-138 in lung cancer (32), miR-181a in lung cancer (128), miR-16-5p in thyroid carcinoma (129), miR-129-5p in laryngeal squamous cell carcinoma (130), miR-199-5p in renal carcinoma (126), and miR-195-5p in osteosarcoma (131).

SNHG12 stimulates EMT in lung cancer by ceRNA targeting of miR-218 (132), and mediates doxorubicin resistance via miR-320a repression and MCL1 (MCL1 apoptosis regulator, BCL2 family member) up-regulation in osteosarcoma (133).

In glioma, SNHG12 targeting of miR-101-2 leads to enhanced cell growth (134), malignant progression via TDP43 (TAR DNA-binding protein 43) (135), and enhanced proliferation/migration capacity due to the association with the Hu antigen R (136).

In cervical cancer, SNHG12 promotes proliferation, invasion, and migration, by targeting miR-424-5p (124). This microRNA acts as a tumor suppressor in cervical cancer, by down-regulation of *CHK1* (checkpoint kinase 1) gene (137) and up-regulation of *aprataxin*, which leads to radiosensitivity (138).

SNHG12 activation of PI3K/AKT signaling pathway in gastric cancer promoted cell proliferation, cell cycle progression, and inhibition of apoptosis (139). SNHG12 stimulation of PI3K/AKT pathway in colorectal cancer leads to cell growth (140). PI3K/AKT is a signaling pathway that becomes activated in response to growth factor stimulation. This signaling pathway is a master regulator of cell activity, and it acts by inhibiting p21, p53, and BAX, while activating the MDM2 oncogene (141, 142).

SNHG12 inhibits miR-320 (125), which acts as a tumor suppressor in gastric cancer, by lowering the expression of FoxM1 and P27^KIP1^ (143).

In hepatocellular carcinoma, SNHG12 induces miR-199a/b-5p underexpression, which leads to the overexpression of *MLK3* (mixed-lineage kinase 3) and the stimulation of the NF-κB pathway (32).

In the non-small cell lung cancer, SNHG12 acts as a ceRNA for miR-138 (144) and miR-181a (128), causing increased proliferation and colony-formation capabilities, as well as impaired apoptosis (128, 144). MiR-138 is an inhibitor of proliferation, autophagy, and metastasis (145). Its down-regulation also leads to chemoresistance (146). MiR-181a hinders lung cancer cell proliferation and migration by targeting *CDK1* (cyclin dependent kinase 1) (147) and *KRAS* (148). According to a recent meta-analysis, miR-181a is majorly linked to lung cancer patient's survival rate (149).

In osteosarcoma, SNHG12 causes increased cell proliferation and migration as well as stimulated angiogenesis, through up-regulation of *AMOT* (angiomotin) (150). In triple negative breast cancer, it also leads to increased cell proliferation, migration, and inhibited apoptosis, through the stimulation of *MMP13* and *SNHG12* genes activated by c-Myc (151).

## Small Nucleolar RNA Host Gene 15 (SNHG15)

The SNHG15 is the origin for SNORA 9 (152).

The main molecular activity of SNHG15 is related to its targeting of miRNAs, as follows: miR-141-3p in hepatocellular carcinoma (153), miR-338-3p in prostate cancer (154), miR-211-3p in lung cancer (155, 156), and miR-211-3p in breast cancer (157). SNHG15 also leads to activation of NF-KB pathway in renal cell carcinoma (158).

SNHG15 is involved in colorectal cancer cell proliferation and migration via miR-141 (159) and miR-338-3p sponging (160), along with up-regulation of AIF (Allograft Inflammatory Factor 1) (152), and activation of transcription factor SLUG (45). In thyroid cancer, SNHG15 is a ceRNA for miR-510-5p (161) and miR-200a-3p (162).

In osteosarcoma, SNHG15 promotes cell proliferation, autophagy, and migration by acting as a ceRNA for miR-141. The involvement of SNHG15 was also responsible for the proliferation, autophagy, and invasion of osteosarcoma cells (163). MiR-141 is a tumor suppressor in osteosarcoma, inhibiting proliferation and activating apoptosis (164). In pancreatic cancer, SNHG15 was found to be up-regulated and to suppress the transcription of P15 and KLF2 (Kruppel Like Factor 2) by binding to EZH2 and the subsequent methylation at the promoter region of the histone 3 (H3K27me3) (165). This lncRNA is also involved in stimulating the proliferation of endothelial cells from glioma. SNHG15 silencing leads to down-regulation of VEGFA (vascular endothelial growth factor A) and CDC42 (cell division cycle 42), both being proangiogenic genes (166). In gastric cancer, this SNHG15 functions as a tumor promoter by up-regulating MMP2 and MMP9, followed by stimulation of proliferation and invasion (167).

In colorectal cancer, a new mechanism for SNHG15 was found: this lncRNA binds to the zinc-finger domain of SLUG and prevents its ubiquitination. The synchronous coexpression of SNHG and SLUG leads to increased colon cancer cell migration and tumorigenesis capacity (45). Further research and details on the role of SNHG15 in malignant diseases are presented in Supplementary Table 7.

## Small Nucleolar RNA Host Gene 16 (SNHG16)

From the introns of SNHG16, the snoRD1A, snoRD1B, and snoRD1C are generated (168). SNHG16 sponges miR-4518 and activates the PI3K/Akt pathway in glioma, thus causing increased tumor cell proliferation and migration (169). In esophageal cancer, SNHG16 maintains cancer cell viability, impairs apoptosis, and enhances cell migration, by means of miR-140 entrapment, which further results in ZEB1 up-regulation (170).

In cervical cancer, the lncRNA SNHG16 is involved in cell proliferation, apoptosis, and migration by down-regulating miR-216 and up-regulating ZEB1 (171).

In breast cancer, SNHG16 is positively associated with increased proliferation rate, apoptosis evasion, and cell cycle progression through ceRNA binding of miR-98, followed by E2F5 up-regulation (172). Moreover, this lncRNA also has the potential to become a biomarker of early-stage pulmonary malignancy, which is very difficult to diagnose with currently available methods (173). In ovarian cancer, SNHG16 increases malignant cell migration capacity through phosphorylation-mediated activation of AKT (AKT serine/threonine kinase 1) and overexpression of MMP9 (174). Further research and details on the role of SNHG16 in malignant disease are presented in Supplementary Table 8.

## Small Nucleolar RNA Host Gene 20 (SNHG20)

The SNHG20 has 2183 nt (175), and its introns encode for SCARNA16 (176). The SNHG20 is up-regulated in colorectal cancer, hepatocellular cancer, lung cancer, ovarian cancer, and breast cancer. This lncRNA was proven in many studies to interact with p21 (Supplementary Table 9).

SNHG20 down-regulates p21 and E-cadherin in ovarian cancer (177), activates STAT6 in hepatocellular carcinoma (178), represses miR140 in laryngeal squamous cell carcinoma (179), modulates ATM (ataxia telangiectasia mutated)–JAK (janus kinase 2)–PD-L1 (programmed death-ligand 1) pathway in esophageal carcinoma (180), up-regulates TGF-B1 in nasopharyngeal carcinoma (181), and enhances EMT and apoptosis via miR-139 -RUNX2 (runt-related transcription factor 2) in osteosarcoma (182, 183). In breast cancer, SNHG20 causes miR-495 sponging (184), while in oral cancer, it induces cell proliferation via down-regulating PCNA and Ki67 (185) and via targeting miR-197/LIN28 axis (186).

SNHG20, like all SNHG transcripts, is involved in many biological processes, such as: cell proliferation, cell cycle progression, tumorigenesis, apoptosis evasion, and resistance to therapy.

In gastric cancer, it inhibits p21 expression (187), it can interact with miR140-5p to induce resistance to therapy (188), or it can sponge miR-495-3p in order to enhance cell proliferation (189). In ovarian cancer, SNHG20 can activate WNT/β-catenin signaling pathway, which promotes cell proliferation (190).

SNHG20 supports glioma cell survival by sponging miR-4486 and up-regulating MDM2-p53 pathway (191), and silencing p21 (192). This lncRNA is also involved in vasculogenic mimicry, a process of pseudo-vascular formation in highly aggressive cancers, via enhancement of ZRANB2/SNHG20/FOX1 axis (193).

SNHG20 promotes gastric cancer by stimulating cancer cell proliferation, cell cycle progression, invasion, and migration. This is done by down-regulating the expression of the p21 and by activating GSK-3β/β-catenin signaling pathway (187). In ovarian cancer, the overexpression of this lncRNAs also leads to enhanced proliferation and invasion through the activation of the WNT/β-catenin signaling pathway (194).

A number of proinvasion proteins such as ZEB1, ZEB2, N-CAD, and VIM are positively correlated with SNHG20, thus leading to stimulated cell cycle progression, proliferation rate, and migration capacity hepatocellular carcinoma (195) and breast cancer (184).

In lung cancer, SNHG20 overexpression causes increased cell proliferation, invasion, and migration capacity, by changing the DNA methylation pattern, after interaction with EZH2 and epigenetic repression of p21 (175). p21 is suppressed by SNHG20 in colorectal cancer, where it stimulates cyclin A1, leading to intensification of proliferation and migration (196) P21 is classically considered as a tumor suppressor, being involved in the downstream pathway of the p53 cell cycle arrest and in the assembly of cyclin D-CDK4/CDK6. However, when p53 is dysregulated or when p21 acquires mutations, it becomes an oncogene (197). Further research and details on the role of SNHG20 in malignant diseases are presented in Supplementary Table 9.

**Figure 2:**
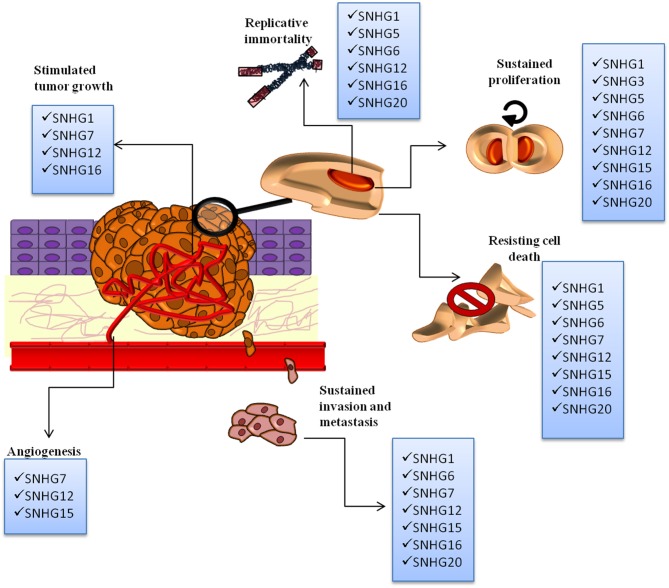
The SNHGs' involvement in the following malignant processes: replicative immortality, sustained proliferation, angiogenesis, resisting cell death, invasion, and metastasis and tumor growth. All SNHGs promote tumor development and progression.

SNHGs are overexpressed from the malignant transformation of a normal cell to the installment of metastasis. They sustain replicative immortality, speeding up of proliferation rate, resistance to programmed cell death, tumor growth, neoangiogenesis (and in some instances vascular mimicry), local invasion, migration, and formation of metastasis. The overall representation of different SNHGs' involvement in cancer initiation and progression is found in Figure 2.

## Future Applications Based on Current Knowledge-Discussions

**Figure 3:**
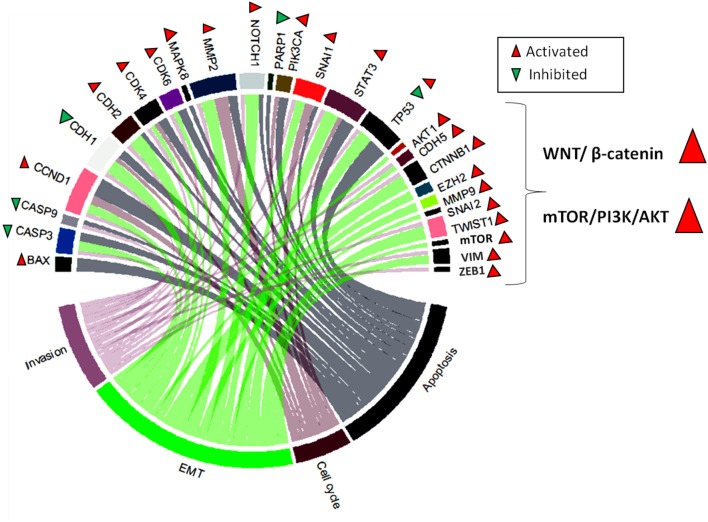
Chord plot representing the most frequently modulated genes by SNHGs. These are: BAX (Bcl-2-associated X protein), CASP3 (caspase 3), CASP9 (caspase 9), CCND1 (cyclin-D1), CDH1 (E-cadherin), CDH2 (N-cadherin), CDK4 (cyclin-dependent kinase 4), CDK6 (cyclin-dependent kinase 6), MAPK8 (mitogen-activated protein kinase), MMP2 (metalloproteinase 2), NOTCH1 (Notch homolog 1), PARP1 (poly [ADP-ribose] polymerase 1), PIK3CA (phosphatidylinositol-4,5-bisphosphate 3-kinase catalytic subunit alpha), SNAI1, STAT3 (signal transducer and activator of transcription 3), TP53, AKT1 (AKT serine/threonine kinase 1), CDH5 (vascular endothelial cadherin), CTNNB1 (beta-catenin), EZH2 (enhancer of zeste 2 polycomb repressive complex 2 subunit), MMP9 (metalloproteinase 9), SNAI2 (snail family transcriptional repressor 2), TWIST1, mTOR, VIM (vimentin), and ZEB1 (zinc finger E-box binding homeobox 1). These are associate in a different degree to the most frequently dysregulated cellular processes in cancer: invasion, EMT, cell cycle, and apoptosis. SNHGs also act by activating two major signaling pathways: WNT/β-catenin and mTOR/PI3K/AKT.

Through our research of literature, we identified that the general activity of SNHGs in malignant disease is related to stimulation of the following malignant processes: EMT, invasion, proliferation, cell cycle progression, and apoptosis evasion. The most common signaling pathways activated by SNHGs are: WNT/β-catenin (67, 198) and mTOR/PI3K/AKT (199–201). SNHGs also influence the NF-kB (202) and Hippo signaling pathways (203); however, more data are needed to include these two pathways in the general mechanisms of SNHG activity (Figure 3).

The oncogenic role of SNHGs in malignancies is supported by solid scientific data. These ncRNAs act on multiple levels to induce a more aggressive phenotype of cancer cell, and their overexpression is correlated with lower overall and progression-free survival. There are a number of meta-analyses that offer additional support in favor of bringing the research of SNHGs into clinical trials. SNHG16 is associated with advanced clinicopathological features and worse overall survival in the case of malignancies located in the lung, ovaries, cervix, bladder, and esophagus (204). SNHG1 has a progressive increase in expression correlated with disease progression in the case of the following tumor localizations: lung, esophagus, bone, brain, stomach, liver, and colon. A high level of SNHG1 is associated with poor overall and disease-free survival in all of these malignancies (205–207). SNHG6 leads to worse prognosis and a propensity of distant metastasis formation (208).

The oncogenic activity of SNHGs can be impaired by the temporary silencing of SNHGs at RNA level with the help of RNA interference (194, 209) or the permanent deletion of these lncRNAs in cancer cells through genome editing techniques (152, 210).

A careful evaluation of the SNHGs inhibition in normal cells would be extremely useful in evaluating the potential druggability of SNHGs. From what we know so far, the downstream effect of SNHG inhibition on their corresponding snoRNAs-miRNAs/piRNAs will be dependent on the type of change: knock out or knockdown. SNHGs are targeted through permanent changes at the DNA level through gene therapy. This is a desired effect if the therapy is carefully monitored to have high specificity for tumor cells and to avoid normal cells. In normal cells, snoRNAs have essential physiological functions in rRNA and mRNA processing. A recent study has already evaluated the ability of the CRISPR/Cas9 to target protospacer adjacent motifs (PAMs) located in the structure of snoRNAs from the same SNHG, GAS5. The induced mutations affected specifically the ability to form secondary Kink-turn structure in each SNORD (SNORD74, SNORD77, and SNORD80). Moreover, the editing of PAM located in the D' box of SNORD75 affected the alternative splicing of SNHG, thus showing that snoRNAs can modulate the alternative splicing of their SNHG of origin by affecting the m6A methyltransferase complex (211). A better alternative to genome editing is the temporal inhibition of SNHGs through siRNAs. This is also the main therapeutic strategy evaluated across various studies. For instance, in renal cell carcinoma, the RNAi silencing of SNHG15, *in vitro*, led to decreased proliferation, invasion, and migration capacity (158). The silencing of SNHG1 in colon cancer cells lowered their malignant potential (212). The knockdown of SNHG6 in glioma causes a reduced growth rate of treated cells (213). The SNHG inhibition can reverse chemotherapeutic resistance, as in the case of sorafenib and SNHG1 (200). However, because of their ability of acting as ceRNAs for miRNAs (214) or being the primary transcript for various snoRNAs, piRNAs, and miRNAs, the down-stream effect of SNHG therapeutic modulation would be very difficult to predict and control, especially in case of non-specific targeting of tumor cells. For instance, we still don't know whether siRNA silencing of SNHGs affects the downstream levels of snoRNAs-piRNAs/miRNAs originated from the targeted SNHG.

## Conclusion

Because of the versatility and function in the majority of cancers, SNHGs are becoming increasingly important for molecular research in cancer. The data on these non-coding RNAs are abundant, and the validation of their role in the progression and severity of malignant diseases has been made clear during the last year. However, a general overlook on these lncRNAs is still lacking as well as a better understanding of their influence at the molecular level. The majority of SNHGs (SNHG1, SNHG12, SNHG20, SNHG15, SNHG16, SNHG3, SNHG5, SNHG6, SNHG7) function by sponging tumor- suppressing microRNAs, allowing the oncogene transcripts to be expressed. The SNHGs can also cause epigenetic alterations in the genome, transcription initiation through interaction with transcription factors, direct binding and up-regulation of mRNAs and extension of protein life through prevention of ubiquitination. SNHGs also activate the signaling pathways commonly involved in cancer development and progression, such as Wnt/β-catenin and mTOR/PI3K/AKT. The SNHGs have the potential to act as cancer biomarkers or even therapeutic targets because of their ability to retain the tumor suppressor microRNAs. The studies on SNHGs increase by the day, and these classes of non-coding RNAs might constitute the next miRNAs in the field of molecular oncology. However, intrapopulation heterogeneity is still a matter of concern in the analysis of every transcript as biomarker and more data are needed considering especially that there are some reports, even if scarce, on the role of these lncRNAs acting as tumor suppressors. More analysis should also be done on regulatory mechanism at DNA and protein level and SNHGs stability maintenance in the disadvantage of snoRNAs, although data present a positive correlation between SNHGs and their corresponding snoRNAs.

## Author Contributions

A-AZ: first co-author and PhD student specializing in molecular biology who wrote the majority of the paper. AT: PhD student in cellular and molecular biology who majorly contributed to the literature review regarding functional *in vitro* assays and tables 1–9 writing. CB: researcher with experience in molecular biology who contributed to the design of figures, general concept of the article, and text writing. CS: MD, PhD expert in global oncology, researcher with multiple specializations in pedriatic oncology, gynecology, and public health who wrote about the clinical applications of SNHGs. CI: surgeon and physician scientist with expertise in surgical oncology, ncRNAs, and molecular profiling of cancers who critically revised the manuscript and wrote part of the introduction. IB-N: Professor of Medical Biotechnology who coordinated the article topic, analysis, and interpretation and was responsible for the final version of the article, including proofreading, writing, and reorganizing the article.

### Conflict of Interest

The authors declare that the research was conducted in the absence of any commercial or financial relationships that could be construed as a potential conflict of interest.
