# Supplementary material for: An Emerging Class of Long Non-coding RNA With Oncogenic Role Arises From the snoRNA Host Genes
Source: Front Oncol. 2020 Apr 7;10:389. doi: 10.3389/fonc.2020.00389 (PMC7154078; doi:10.3389/fonc.2020.00389)
Supplement: Supplementary file 1 [file Table_1.docx]

# Tables

Table S1: The studies of SNHG1 role in solid tumors. T=tumor tissue , C=cells, X=no mention.

| Type of cancer | Biological role | Molecular mechanism | Cells | Tissue | Ref. |
| --- | --- | --- | --- | --- | --- |
| Colorectal cancer | Cell growth, apoptosis, invasion, migration | Up-regulation of BCL-2  Down-regulation of BAX, BID, BIM, MMP7, Cyclin D, TCGF7  Activation of WNT pathway | C | x | (Qi et al., 2017) |
| Colorectal cancer | Cell proliferation and viability, cell cycle progression | Down-regulation of p53, p21 and BAX | C | x | (Zhao et al., 2018c) |
| Colorectal cancer | Tumor growth, cell proliferation | miR-145 sponging  Up-regulation of p70S6K and E2F3 | C | x | (Tian et al., 2018) |
| Esophageal cancer | Cell proliferation, invasion capacity and EMT | Down-regulation of HES-, Notch1, Vimentin and N-cadherin  Up-regulation of E-cadherin | C | T | (Zhang et al., 2017) |
| Esophageal cancer | N/A | miR-338 sponging,  Up-regulation of CST3 | C | x | (Yan et al., 2017b) |
| Glioma | Prognosis, cell proliferation, invasion, and apoptosis | N/A | C | x | (Wang et al., 2017e) |
| Hepatocellular carcinoma | Cell proliferation, invasion, and migration | Sponging of miR-195 | C | T | (Zhang et al., 2016b) |
| Hepatocellular carcinoma | Larger tumor size, poor differentiation, cell proliferation, cell cycle progression, apoptosis | Inhibition of p53 | C | T | (Zhang et al., 2016d) |
|  |  | Up-regulation of BAX, FAS, and CDKN1A | C | T |  |
| Laryngeal squamous cell carcinoma | Cell proliferation, invasion and migration | Up-regulation of BCL-2, SNAIL, VIM, MMP2, MMP9  Down-regulation of E-CAD, BAX | C | T | (Lin et al., 2018) |
| Lung cancer | Cell proliferation | N/A | C | x | (You et al., 2014) |
| Lung cancer | Cell proliferation | miR-101 sponging | C | T | (Cui et al., 2017) |
|  |  | SOX9 up-regulation | C | T |  |
|  |  | Wnt/β-catenin signaling pathway activation | C | T |  |
| Lung cancer | Cell viability, proliferation, migration, and invasion | miR-145 sponging  Up-regulation of MTDH | C | x | (Lu et al., 2018a) |
| Nasopharyngeal carcinoma cell | Invasion and migration | miR-145a sponging  Up-regulation of NUAK1 | C | T | (Lan and Liu, 2019) |
| Neuroblastoma | Increased cell viability | miR-15b sponging  Up-regulation of SIAH1 | C | x | (Chen et al., 2018) |
| Osteosarcoma | Cell proliferation, cell migration and EMT | miR-577 sponging | C | x | (Jiang et al., 2018c) |
|  |  | WNT2B/Wnt/β-catenin pathway activation | C | x |  |
| Osteosarcoma | Cell proliferation, apoptosis, tumor growth, migration and invasion | miR-326 sponging | C | T | (Wang et al., 2018a) |
|  |  | Up-regulation of NOB1 | C | T |  |
| Ovarian carcinoma | Cell proliferation, self-renewal capacity, apoptosis, invasion and metastasis | Up-regulation of N-CAD, VIM, MMP-2, MMP-9  Down-regulation of E-CAD | C | T | (Ge et al., 2018) |
| Prostate cancer | Cell proliferation | miR-199a sponging | C | T | (Li et al., 2017) |
|  |  | Up-regulation of CDK7 | C | T |  |

**Table S2:** The studies of SNHG3 role in solid tumors, T=tumor tissue , C=cells, X=no mention

| Type of cancer | Biological role | Molecular mechanism | Cells | Tissue | Ref. |
| --- | --- | --- | --- | --- | --- |
| Breast cancer | Malignant development | Higher SNHG3 level in ER/PR negative tumors | C | T | (Taherian-Esfahani et al., 2019) |
| Colorectal cancer | Cell proliferation | miR-182 sponging  Up-regulation of c-Myc, CCNB1, CCND2, CDK4, and E2F1 | C | x | (Huang et al., 2017) |
| Glioma | Malignant progression | Epigenetic silencing of KLF2 and p21 | C | T | (Fei et al., 2018) |
| Hepatocellular carcinoma | Tumor size, tumor relapse | N/A | C | T | (Zhang et al., 2016e) |
| Hepatocellular carcinoma | Drug resistance | miR-128 sponging  Up-regulation of CD151 | C | x | (Zhang et al., 2019f) |
| Laryngeal carcinoma | Cell proliferation and migration | miR-384 sponging  Up-regulation of WEE1 | C | T | (Wang et al., 2019e) |
| Lung cancer | Cell proliferation | N/A | C | x | (Liu et al., 2018a) |
| Osteosarcoma | Cell growth | miR-196a-5p sponging | C | x | (Chen et al., 2019b) |
| Osteosarcoma | Cell invasion and migration | miR-151a-3p sponging  Up-regulation of RAB22A | C | T | (Zheng et al., 2019) |
| Ovarian cancer | Malignant progression | N/A | C | x | (Hong et al., 2018) |
| Ovarian cancer | Energy metabolism | miR-186a sponging  Up-regulation of PDHB, PKM, IDH2, UQCRH | C | x | (Li et al., 2018b) |

**Table S3:** The studies of SNHG5 role in solid tumors. T=tumor tissue , C=cells, X=no mention

| Type of cancer | Biological role | Molecular mechanism | Cells | Tissue | Ref. |
| --- | --- | --- | --- | --- | --- |
| Acute myeloid leukemia | Possible biomarker | N/A | C | x | (Li and Sun, 2018) |
| Bladder cancer | Cell proliferation, cell cycle progression, apoptosis | p27 down-regulation | C | T | (Ma et al., 2018) |
| Breast cancer | Cell proliferation | miR-154-5p sponging  Up-regulation of PCNA | C | x | (Chi et al., 2019a) |
| Chronic myeloid leukemia | Chemoresistance | miR-205-5p sponging  Up-regulation of ABCC2 | C | x | (He et al., 2017) |
| Colorectal cancer | Cell cycle progression, apoptosis | Increased stability of SPATS2 mRNA | C | x | (Damas et al., 2016) |
| Colorectal cancer | Cell proliferation, metastasis | miR-132-3p sponging  Up-regulation of CREB5 | C | T | (Zhang et al., 2019e) |
| Gastric cancer | Apoptosis, drug resistance | Up-regulation of BCL-2  Down-regulation of BAX | C | x | (Li et al., 2019d) |
| Gastric cancer | Autophagy | METase mediated overexpression of SNHG5  miR-20a sponging  Up-regulation of ATG7 | C | T | (Xin et al., 2019) |
| Gastric cancer | Cell proliferation and migration | miR-32 sponging | C | x | (Zhao et al., 2017) |
| Gastric cancer | Cell proliferation and migration | Up-regulation of KLF4 | C | x |  |
| Glioma | Cell cycle progression | miR-205 sponging  Up-regulation of E2F3 | C | x | (Li et al., 2019g) |
| Glioma | Cell proliferation, invasion and tumorigenesis | Wnt/CTNNB1 pathway activation | C | x | (Hu et al., 2019) |
| Hepatocellular carcinoma | Cell cycle progression, apoptosis, invasion | miR-26a-5p sponging  Up-regulation of GSK3B, MMP-2, MMP-9, BCL-2, CDK4, CDK6  Down-regulation of BAX | C | x | (Li et al., 2018c) |
| Lung cancer | Drug resistance | miR-377 sponging  Up-regulation of: CASP1 | C | x | (Wang et al., 2018c) |
| Melanoma | Cell growth | miR-26a-5p sponging  Up-regulation of TRPC3 | C | T | (Gao et al., 2019a) |
| Osteosarcoma | Apoptosis | miR-212-3p sponging  Up-regulation of SGK3, Casp-3, Caps-9, PARP | C | x | (Ju et al., 2018) |
| Osteosarcoma | Tumorigenesis | miR-26a sponging  Up-regulation of ROCK1, MLC and MYPT | C | x | (Wang et al., 2018d) |
| Ovarian cancer | Cell proliferation, metastasis | N/A | C | x | (Zhao and Fan, 2019) |

**Table S4:** The studies of SNHG6 role in solid tumors. T=tumor tissue , C=cells, X=no mention T=tumor tissue and C=cells

| Type of cancer | Biological role | Molecular mechanism | Cells | Tissue | Ref. |
| --- | --- | --- | --- | --- | --- |
| Bladder cancer | EMT | miR-125b sponging  Up-regulation of SNAIL1/2, NUAK1 | C | x | (Wang et al., 2019b) |
| Breast cancer | Cell proliferation | N/A | C | T | (Jafari-Oliayi and Asadi, 2019) |
| Breast cancer | Cell proliferation | miR-26a sponging  Up-regulation of VASP | C | T | (Li et al., 2019b) |
| Breast cancer | Cell proliferation, migration and invasion | miR-26a-5p sponging  Up-regulation of:MAPK6 | C | T | (Lv et al., 2019) |
| Breast cancer/lung cancer | Possible biomarker | N/A | x | x | (Ding et al., 2017) |
| Colorectal cancer | Malignant progression, cell invasion | miR-181a-5p sponging  Up-regulation of E2F5, N-CAD, VIM  Down-regulation of E-CAD | C | T | (Yu et al., 2019a) |
| Colorectal cancer | Cell migration and invasion | miR-26a sponging  Up-regulation of EZH2, N-CAD, VIM, SNAIL  Down-regulation of E-CAD | C | T | (Zhang et al., 2019d) |
| Colorectal cancer | Cell proliferation, cell cycle progression | miR-26a/b and miR-214 sponging  Up-regulation of: Cyclin D, CDK4, CDK6, EZH2  Down-regulation of: p14, p15, p16, E-CAD | C | x | (Xu et al., 2019c) |
| Colorectal cancer | Cell proliferation | miR-760 sponging  Up-regulation of FOXC1 | C | T | (Zhu et al., 2018c) |
| Colorectal cancer | Cell proliferation and invasion | miR-101-3p sponging  Down-regulation of UPF1, E-CAD  Up-regulation of ZEB1, N-CAD, VIM, SNAIL, SLUG, MMP2, MMP9  Activation of SMAD2 and SMAD3, with no effect on their expression level | C | T | (Wang et al., 2019i) |
| Colorectal cancer | Cell proliferation and metastasis | Inhibition of AKT, PI3K  mTOR activation (no effect on their protein level)  Up-regulation of ETS1 | C | T | (Meng et al., 2019) |
| Colorectal cancer | Cell proliferation, cell cycle progression and apoptosis | N/A | C | T | (Li et al., 2018a) |
| Colorectal cancer | N/A | Up-regulation of EZH2  Inhibition of p21 transcription through epigenetic modulation | C | x | (Li et al., 2018f) |
| Colorectal cancer | Possible biomarker | N/A | x | x | (Ansari et al., 2019) |
| Colorectal cancer | Possible biomarker | N/A | x | x | (Xue et al., 2017) |
| Esophageal squamous cell carcinoma | Cell proliferation and apoptosis | N/A | C | T | (Fan et al., 2018) |
| Esophageal squamous cell carcinoma | Cell proliferation, migration and invasion | N/A | C | T | (Zhang et al., 2019g) |
| Gastric cancer | Cell invasion and migration | miR-101 sponging  Down-regulation of : E-CAD, β-catenin, p27  Up-regulation: N-CAD, VIM, ZEB1 | C | T | (Yan et al., 2017a) |
| Gastric cancer | Cell proliferation | Inhibition of JNK, p38, ERK1/2  Down-regulation of p53, p21  Up-regulation of EZH2 | C | x | (Li et al., 2018d) |
| Glioma | Cell proliferation | Down-regulation of: p21  Inhibition of: CASP3, CASP9 | C | T | (Cai et al., 2018) |
| Glioma | Cell proliferation, migration and invasion | miR-101-3p sponging | C | T | (Meng et al., 2018) |
| Hepatocellular carcinoma | Cell cycle progression, apoptosis | miR-101 sponging | C | T | (Chang et al., 2016) |
|  |  | Up-regulation of SNORD87, c-Myc, CDK4, CDK6, CCND1, BAX, MMP9, MMP2, VIM, Fibronectin, ZEB1  Down-regulation of E-CAD, Claudin-1  Inhibition of CASP3, PARP | C | T |  |
| Hepatocellular carcinoma | Cell proliferation | miR-139-5p sponging  Up-regulation of SERPINH1 | C | T | (Wu et al., 2019a) |
| Hepatocellular carcinoma | Methylation | Lower general DNA methylation through inhibition of S-adenosylmethionine  miR-1297 sponging  Down-regulation of MAT1A | C | T | (Guo et al., 2018b) |
| Lung cancer | Cell proliferation and EMT | Up-regulation of N-CAD, VIM, E2F7  Down-regulation of E-CAD, Zo-1  miR-26a-5p sponging | C | x | (Liang et al., 2018) |
| Osteosarcoma | Autophagy | miR-26a sponging  Down-regulation of ULK1  Inhibition of CASP3, ATF3 | C | T | (Zhu et al., 2019b) |
| Osteosarcoma | Cell proliferation | Up-regulation of p21 and KLF2  Down-regulation of CCND2 | C | T | (Ruan et al., 2018) |
| Ovarian cancer | Cell proliferation and migration | miR-4465 sponging  Up-regulation of N-CAD, MMP2, MMP9, EZH2 | C | T | (Wu et al., 2019d) |
| Prostate cancer | N/A | N/A | C | x | (Yan et al., 2019) |
| Renal cancer | Metastasis | N/A | C | T | (An et al., 2018) |
| Urinary system tumor | Cell proliferation, migration and invasion | miR-15a sponging  Down-regulation of p53,  Up-regulation of CCND2  Inhibition of CASP3, CASP9, TAK, JNK | C | x | (Su et al., 2019a) |

**Table S5:** The studies of SNHG7 role in solid tumors. T=tumor tissue , C=cells, X=no mention

| Type of cancer | Biological role | Molecular mechanism | Cells | Tissue | Ref. |
| --- | --- | --- | --- | --- | --- |
| Bladder cancer | Cell proliferation | Indirect increase of BAX, p21 and E-CAD | C | T | (Xu et al., 2019b) |
| Bladder cancer | Cell proliferation and migration | Wnt/B-catenin pathway activating  Up-regulation of β-catenin, CCND2, c-Myc  Down-regulation of E-CAD | C | T | (Chen et al., 2019d) |
| Bladder cancer | Cell proliferation, invasion and apoptosis | Up-regulation of N-CAD, VIM, Snail,  Down-regulation of E-CAD | C | T | (Zhong et al., 2018) |
| Breast cancer | Cell cycle progression, iinvasion and migration | miR-34a sponging  Up-regulation of MMP2, MMP7, VIM, Snail, Notch-1, Survivin, CCND1  Down-regulation of E-CAD | C | T | (Sun et al., 2019c) |
| Breast cancer | Cell proliferation, metastasis | miR-186 sponging | C | T | (Luo et al., 2018) |
| Breast cancer | Cell proliferation and invasion | miR-381 sponging | C | x | (Gao and Zhou, 2019) |
| Colorectal cancer | Cell proliferation | miR-34a sponging  Up-regulation of GALNT1  Activation of PI3K, Akt, mTOR | C | T | (Li et al., 2018e) |
| Colorectal cancer | Cell proliferation and metastasis | Up-regulation of GALNT1  miR-216b sponging | C | T | (Shan et al., 2018) |
| Esophageal cancer | Cell proliferation and apoptosis | Up-regulation of p15 and p16 | C | T | (Xu et al., 2018b) |
| Gastric cancer | Cell proliferation and apoptosis | Inhibition of p15 and p16 | C | T | (Wang et al., 2017b) |
| Gliobastoma | Cell proliferation, migration and invasion | miR-5095 sponging  Wnt/β-catenin signaling pathway activation | C | T | (Ren et al., 2018) |
| Hepatocellular carcinoma | Metastasis | Down-regulation of RBM5 | C | T | (Sun et al., 2019a) |
| Hypopharyngeal cancer | Cell growth | N/A | C | x | (Wu et al., 2019c) |
| Lung cancer | Cell proliferation, apoptosis, invasion and migration | Up-regulation of *FAIM2* | C | x | (She et al., 2016) |
| Lung cancer | Cell proliferation, apoptosis, invasion and migration | miR-193b sponging  Up-regulation of FAIM2 | C | T | (She et al., 2018) |
| Melanoma | Cell migration | Up-regulation of SOX4 | C | T | (Zhang et al., 2019b) |
| Nasopharyngeal carcinoma | Cell proliferation and invasion | Up-regulation of ROCK1 | C | T | (Wang et al., 2019f) |
| Neuroblastoma | Cell cycle progression, invasion, migration | Up-regulation of β-catenin, VIM, N-CAD, STAT2, STAT3  Down-regulation of E-CAD  miR-653 sponging | C | T | (Chi et al., 2019b) |
| Osteosarcoma | Cell growth and EMT | Regulation of miR-34a  Up-regulation of: Notch1, BCL-2, CDK6, SMAD4 | C | T | (Deng et al., 2018) |
| Osteosarcoma | Cell proliferation | Down-regulation of:p53 via binding DNMT1 | C | T | (Zhang et al., 2019c) |
| Pancreatic cancer | Cell proliferation | miR-342-3p sponging  Up-regulation of ID4 | C | T | (Cheng et al., 2019) |
| Prostate cancer | Cell proliferation, cell growth, angiogenesis | miR-503 sponging  Up-regulation of: Cyclin D1, CDK4, CDK6 | C | T | (Qi et al., 2018) |
| Prostate cancer | EMT | Up-regulation of N-CAD, WNT2B  Down-regulation of E-CAD  miR-324 sponging | C | T | (Han et al., 2019b) |
| Renal cancer | N/A | N/A | C | T | (He et al., 2016) |
| Thyroid cancer | Cell proliferation and apoptosis | Up-regulating of BDNF | C | T | (Wang et al., 2019k) |

**Table S6:** The studies of SNHG12 role in solid tumors. T=tumor tissue , C=cells, X=no mention

| Type of cancer | Biological role | Molecular mechanism | Cells | Tumor tissue | Ref. |
| --- | --- | --- | --- | --- | --- |
| Natural killer/T-cell lymphoma | Cell proliferation and drug resistance | Its transcription is up-regulated by c-Myc activity | C | x | (Zhu et al., 2019a) |
| Bladder cancer | Cell proliferation | Up-regulation of HDAC10, AGER  Down-regulation of PCDH7, LATS2 | C | x | (Jiang et al., 2018a) |
| Breast cancer | Cell proliferation, apoptosis and migration | Up-regulation of MMP13  Its transcription is up-regulated by c-Myc activity | C | T | (Wang et al., 2017c) |
| Cervical cancer | Cell proliferation, migration and invasion | miR-424-5p sponging | C | T | (Dong et al., 2018a) |
| Cervical cancer | Cell proliferation, migration and invasion | miR-125b sponging  Up-regulation of STAT3 | C | x | (Jin et al., 2019) |
| Colorectal cancer | Cell proliferation and invasion | miR-16 sponging | C | T | (Liu et al., 2019f) |
| Colorectal cancer | Proliferation, cell cycle arrest, apoptosis | Up-regulation of CDK4, CDK6 and CCND1  Inhibition of CASP3  Activation of AKT | C | x | (Wang et al., 2017a) |
| Gastric cancer | Cell proliferation and metastasis | miR-199a/b-5p sponging | C | x | (Yang et al., 2018a) |
| Gastric cancer | Cell proliferation and migration | miR-16 sponging | C | x | (Zhao et al., 2019) |
| Gastric cancer | Larger tumor size, tumor node metastasis stage, distant metastasis, lymphatic metastasis, cell growth, colony formation, proliferation and invasion | miR-320 sponging  Up-regulation of: CRLK  Activation of AKT, ERK | C | x | (Zhang and Lu, 2018) |
| Glioma | Cell growth | miR-101-2 sponging  Up-regulation of: FOXP1 | C | x | (Sun et al., 2018b) |
| Glioma | Cell proliferation and migration | Associated with Hu antigen R (HuR) | C | x | (Lei et al., 2018) |
| Glioma | Malignant progression | TDP43 stabilizes SNHG12  miR-195-sponging  Up-regulation of SOX5 | C | T | (Liu et al., 2018b) |
| Laryngeal squamous cell carcinoma | Cell proliferation, invasion and apoptosis | miR-129-5p sponging  Up-regulation of WWP1 (WT) | C | x | (Li et al., 2019a) |
| Lung cancer | Cell proliferation, migration and invasion | miR-218 sponging  Inhibition of CASP3, CASP9  Up-regulation of MMP9, VIM, SLUG, ZEB2  Down-regulation of E-CAD | C | x | (Wang et al., 2019j) |
| Lung cancer | Cell proliferation, self-renewal capacity. apoptosis, drug resistance | miR-138 sponging | C | x | (Wang et al., 2017f) |
| Lung cancer | Cell proliferation, self-renewal capacity. apoptosis, drug resistance | miR-181a sponging  Up-regulation of SLUG  Activation of MAPK1, MAP2K1 | C | x | (Wang et al., 2017d) |
| Lung cancer | Receptor-mediated endocytosis,  macropinocytosis, and phagocytosis | N/A | x | T | (Lei et al., 2019) |
| Nasopharyngeal carcinoma | Cell proliferation, migration and invasion | Up-regulation of N-CAD, VIM, NOTCH1, P21, HES1  Down-regulation of E-CAD | C | x | (Liu et al., 2018c) |
| Osteosarcoma | Cell proliferation, migration, and angiogenesis | Up-regulation of AMOT | C | x | (Ruan et al., 2016) |
| Osteosarcoma | Drug resistance | miR-320a sponging  Up-regulation of MCL1 | C | x | (Zhou et al., 2018a) |
| Osteosarcoma | Tumorigenesis and metastasis | Up-regulation of CDK4, CDK6, CCND1, NOTCH-2  miR195-5p sponging | C | x | (Zhou et al., 2018b) |
| Ovarian carcinoma | Cell proliferation and migration | miRNA-129 sponging  Up-regulation of SOX4 | C | x | (Sun and Fan, 2019) |
| Papillary thyroid carcinoma | Cell growth and invasion | miR-16-5p sponging  Up-regulation of MMP13, MMP9, BCL-2, PCNA  Down-regulation of BAX | C | x | (Feng et al., 2019) |
| Papillary thyroid carcinoma | Cell proliferation and migration | Up-regulation of β-catenin, CCND1, MMP2 | C | x | (Ding et al., 2018) |
| Prostate cancer | Cell proliferation, invasion and migration | miRNA-195 sponging  Wnt/b-catenin signaling pathway activation | C | x | (Song et al., 2019) |
| Prostate cancer | Tumorigenesis, cell growth, migration, and invasion | miR-133b sponging | C | x | (Cheng et al., 2020) |
| Renal cell carcinoma | Cell viability, migration, tumor growth | miR-199a-5p sponging  Up-regulation of HIF1α  Repression of PARP cleavage | C | x | (Chen et al., 2019c) |

**Table S7:** The studies of SNHG15 role in solid tumors. T=tumor tissue , C=cells, X=no mention

| Type of cancer | Biological role | Molecular mechanism | Cells | Tissue | Ref. |
| --- | --- | --- | --- | --- | --- |
| Breast cancer | Cell proliferation, migration and invasion | miR-211-3p sponging  Up-regulation of PCNA, CCND1, VIM, MMP2, MMP9, SNAI1  Down-regulation of BAX, E-CAD  Inhibition of CASP3 cleavage | C | x | (Kong and Qiu, 2018) |
| Colon cancer | Cell proliferation | Impairs β-transducin repeat containing (BTRC)- mediated ubiquitination of SLUG | C | x | (Jiang et al., 2018b) |
| Colorectal cancer | Cell proliferation | Down-regulation of BAX, E-CAD, WNT1, c-MYC, Cyclin D, β-catenin  Up-regulation of N-CAD, VIM, SNAIL  Inhibition of CASP3, CASP9  miR-141 sponging | C | T | (Sun et al., 2019b) |
| Colorectal cancer | Cell proliferation | miR-338-3p sponging  Up-regulation of: FOS, RAB14 | C | T | (Li et al., 2019c) |
| Colorectal cancer | Cell proliferation, invasion and drug resistance | Interaction with AIF (Apoptosis-inducing factor) | C | x | (Saeinasab et al., 2019) |
| Colorectal cancer | Migration | Interaction with β-transducin repeat containing (BTRC) E3, which suppresses ubiquitination of SLUG protein | C | x | (Jiang et al., 2018b) |
| Colorectal cancer (CRC) and colorectal liver metastasis (CLM) | Initiation and progression of CRC and CLM | N/A | C | x | (Huang et al., 2019) |
| Gastric cancer | Cell proliferation and invasion | Up-regulation of MMP2, MMP9 | C | T | (Chen et al., 2016) |
| Glioblastoma | Angiogenesis | miR-153 sponging  Up-regulation of VEGFA, Cdc42 | C | x | (Ma et al., 2017a) |
| Hepatocellular carcinoma | Cell proliferation, migration and invasion | miR-141-3p sponging  Up-regulation of ZEB2 and E2F3 | C | T | (Ye et al., 2019a) |
| Hepatocellular carcinoma | Cell invasion and migration | N/A | x | T | (Zhang et al., 2016c) |
| Lung cancer | Cell proliferation | miR-211-3p sponging  Up-regulation of ZNF217 | C | x | (Ma et al., 2019) |
| Lung cancer | Cell proliferation and metastasis | miR-486 sponging  Up-regulation of CDK14 | C | T | (Jin et al., 2018) |
| Lung cancer | Cell proliferation and invasion | N/A | C | T | (Dong et al., 2018b) |
| Lung cancer | Cell proliferation and migration | miR-211-3p sponging | C | T | (Cui et al., 2018) |
| Osteosarcoma | Cell proliferation, migration, and drug resistance | miR-141 sponging  Up-regulation of ZEB2 and E2F3 | C | x | (Liu et al., 2017c) |
| Ovarian cancer | Cell proliferation, migration, invasion, drug resistance | N/A | C | x | (Qu et al., 2019) |
| Pancreatic cancer | Cell proliferation and apoptosis | Inhibition of: CASP3, PARP  Up-regulation of:CDK2, CDK4  Down-regulation of: P15 and KLF2 | C | T | (Ma et al., 2017b) |
| Pancreatic cancer | Malignant progression | N/A | x | T | (Guo et al., 2018c) |
| Prostate cancer | Malignant progression | miR-338-3p sponging  Up-regulation of N-CAD, FKBP1A  Down-regulation of E-CAD | C | x | (Zhang et al., 2019h) |
| Renal cell carcinoma | Cell proliferation and EMT | Up-regulation of N-CAD, VIM, SNAIL, SLUG, ZEB1  Down-regulation of E-CAD | C | T | (Du et al., 2018) |
| Thyroid cancer | Cell growth and migration | miR-200a-3p sponging  Up-regulation of E-CAD, β-catenin, YAP1  Down-regulation of VIM, N-CAD, MST1, LATS1 | C | T | (Wu et al., 2018) |
| Thyroid cancer | Cell proliferation, migration and invasion | miR-510-5p sponging | C | T | (Liu et al., 2019e) |
| Thyroid cancer | Tumor suppressor | N/A | C | x | (Liu et al., 2019d) |

**Table S8:** The studies of SNHG16 role in solid tumors. T=tumor tissue , C=cells, X=no mention

| Type of cancer | Biological role | Molecular mechanism | Cells | Tissue | Ref. |
| --- | --- | --- | --- | --- | --- |
| Acute lymphoblastic leukemia | Tumor suppressor | miR-124-3p sponging | C | x | (Yang et al., 2019) |
| Bladder cancer | Cell cycle progression, apoptosis | It interacts witch EZH2 and causes methylation of p21 gene | C | T | (Cao et al., 2018) |
| Bladder cancer | Cell proliferation | miR-98 sponging  Up-regulation of BCL2, VIM, N-CAD and SNAIL, c‐Myc, CCND1, andβ‐catenin  Down-regulation of BAX, p27Kip  Inhibition of CASP3, CASP9 | C | T | (Feng et al., 2018) |
| Bladder cancer | EMT | miR-17-5p sponging  Up-regulation of BCL-2, TIMP3  Down-regulation of BAX, CASP3 | C | T | (Peng and Li, 2019) |
| Breast cancer | Cell proliferation and apoptosis | miR-98 sponging  Up-regulation of E2F5 | C | T | (Cai et al., 2017) |
| Cervical | Cell proliferation, apoptosis and migration | miR-216 sponging  Up-regulation of ZEB1 | C | T | (Zhu et al., 2018b) |
| Colorectal cancer | Cell proliferation | miR-200a-3p sponging  Up-regulation of VIM, α-SMA, beta catenin  Down-regulation of E-CAD | C | T | (Li et al., 2019j) |
| Esophageal squamous cell carcinoma | Cell proliferation | Up-regulation of beta-catenin, CCND1, c-MYC | C | T | (Han et al., 2018) |
| Esophagus | Cell viability, apoptosis, migration | miR-140 sponging  Up-regulation of ZEB1 | T |  | (Zhang et al., 2018b) |
| Gastric cancer | Cell proliferation | miR-628-3p sponging  Up-regulation of NRP1 | C | x | (Pang et al., 2019) |
| Gastric cancer | Cell proliferation | miR-135a sponging  Activation of JAK, STAT3 | C | T | (Wang et al., 2019h) |
| Gastric cancer | Cell proliferation, growth, invasion and migration | N/A | C | T | (Lian et al., 2017) |
| Glioma | Apoptosis | miR-4518 sponging  Up-regulation of BCL-2, BC:-xL, MCL-1, PRMT5  Down-regulation of BAX  Activation of AKT, PI3K,  Inhibition of CASP3 | C | x | (Lu et al., 2018b) |
| Glioma | Cell proliferation, increased tumor volume | miR-20a-5p sponging  Up-regulation of E3F1 | C | T | (Yang et al., 2018b) |
| Glioma | Cell proliferation | Down-regulation of CASP3, CASP9, p21  Up-regulation of CCND1, CCNB1 | C | T | (Zhou et al., 2019a) |
| Glioma | Cell proliferation | USF1 up-regulates SNHG16  miR-212-3p sponging  Up-regulation of EphA2, VE-CAD, ALDH1A1 | C | T | (Wang et al., 2019d) |
| Glioma | Malignant progression | miR-373 sponging  Up-regulation of: MMP2, MMP9  Activation of: PI3K, AKT, EGFR | C | x | (Zhou et al., 2019b) |
| Hemangioma | Cell proliferation | miR-520d-3p sponging  Inhibition of CASP3, CASP9  Up-regulation of STAT3 | C | T | (Zhao et al., 2018b) |
| Hepatocellular carcinoma | Cell proliferation | miR-302a-3p sponging  Up-regulation of FGF19 | C | x | (Li et al., 2019f) |
| Hepatocellular carcinoma | Cell proliferation and invasion | miR-186 sponging  Up-regulation of ROCK1 | C | T | (Chen et al., 2019a) |
| Hepatocellular carcinoma | Cell proliferation, invasion and tumorigenesis | miR-195 sponging  Up-regulation of Ki67, MMP2, MMP9 | C | T | (Xie et al., 2019) |
| Hepatocellular carcinoma | Cell proliferation, invasion and tumorigenesis | Up-regulation of p62, mTOR  Activation of p70S6, mTOR, NF-kB | C | x | (Zhong et al., 2020) |
| Hepatocellular carcinoma | Drug resistance | N/A | C | T | (Guo et al., 2019b) |
| Hepatocellular carcinoma | Drug resistance | miR-140-5p sponging | C | T | (Ye et al., 2019b) |
| Hepatocellular carcinoma | Tumorigenesis | miR-4500 sponging  Activation of STAT3  Up-regulation of N-CAD  Down-regulation of E-CAD | C | T | (Lin et al., 2019) |
| Hepatocellular carcinoma | Cell proliferation and drug resistance | miR-93 sponging | C | T | (Xu et al., 2018a) |
| Lung cancer | Cell proliferation and migration | miR-146a sponging  Up-regulation of PNCA, MMP2, MMP9 | C | x | (Han et al., 2019a) |
| Neuroblastoma | Cell proliferation, cell cycle progression | N/A | C | x | (Yu et al., 2019c) |
| Oral carcinoma | Cell proliferation | c-Myc induced up-regulation of SNHG16  Up-regulation of PNCA, MMP2, MMP9, N-CAD, SNAIL,  Down-regulation of E-CAD  Inhibition of CASP3 | C | T | (Li et al., 2019e) |
| Osteosarcoma | Cell migration and invasion | miR-340 sponging | C | T | (Su et al., 2019b) |
| Osteosarcoma | Cell proliferation | miR-205 sponging  Inactivation of CASP3, PARP  Up-regulation of ZEB1 | C | T | (Zhu et al., 2018a) |
| Osteosarcoma | Cell proliferation, migration and invasion | miR-1301 sponging  Up-regulation of BCL9 | C | x | (Wang et al., 2019g) |
| Osteosarcoma | Cell survival and proliferation | miR-98-5p sponging  Up-regulation of STAT3, ZEB1, E2F5 | C | T | (Liao et al., 2019) |
| Osteosarcoma | Drug resistance | miR-16 sponging  Up-regulation of ATG4B | C | T | (Liu et al., 2019c) |
| Ovarian | Cell migration | Up-regulation of MMP9 | C | T | (Yang et al., 2018c) |
| Pancreatic cancer | Tumor growth | miR-218-5p sponging  Up-regulation of HMGB1 | C | T | (Liu et al., 2019b) |
| Retinoblastoma | Cell proliferation | miR-140-5p sponging | C | T | (Xu et al., 2019a) |
| Thyroid cancer | Cell proliferation and invasion | miR-497 sponging  Up-regulation of BDNF, YAP | C | T | (Wen et al., 2019) |

**Table S9:** The studies of SNHG20 role in solid tumors. T=tumor tissue , C=cells, X=no mention

| Type of cancer | Biological role | Molecular mechanism | Cells | Tissue | Ref. |
| --- | --- | --- | --- | --- | --- |
| Breast cancer | Cell proliferation, invasion and migration | miR-495 sponging  Up-regulation of MMP2, MMP9  Activation of beta-catenin, c-MYC | C | x | (Zhao et al., 2018a) |
| Cervical cancer | Cell proliferation and invasion | miR-140-5p sponging  Up-regulation of ADAM10, which further activates p38, ERK, MEK | C | x | (Guo et al., 2018a) |
| Colorectal cancer | Cell proliferation, migration and apoptosis | Down-regulation of p21  Up-regulation of cyclin A1 | C | x | (Li et al., 2016) |
| Esophageal squamous cell carcinoma | Cell growth and metastasis | Up-regulation of N-CAD, VIM, ZEB1  Down-regulation of E-CAD  Activation of ATM, JAK, PD-L1 | C | x | (Zhang et al., 2019a) |
| Gastric cancer | Cell proliferation and invasion | miR-495-3p sponging  Up-regulation of ZFX | C | x | (Cui et al., 2019) |
| Gastric cancer | Cell proliferation, invasion, migration, and cell cycle progression | Direct binding to the EZH2 at protein level  Up-regulation of TWIST1, VIM, β-catenin, GSK-3β  Down-regulation of E-CAD, p21 | C | x | (Liu et al., 2017a) |
| Gastric cancer | Drug resistance | miR140-5p sponging  Up-regulation of NDRG3 | C | x | (Yu et al., 2019b) |
| Glioblastoma | Tumorigenesis and cancer stemness | Up-regulation of CD44, CD133 and Oct-4  Activation of PI3K, AKT and mTOR  Inhibition of CASP3, CASP9 | C | x | (Gao et al., 2019c) |
| Glioma | Cell proliferation | Down-regulation of p21  Up-regulation of CCNA1 | C | T | (Li et al., 2019i) |
| Glioma | Angiogenesis | ZRANB2 stabilizes SNHG20  It mediates FOXK1 degradation through Staufen1 (STAU1)-mediated mRNA decay (SMD)  Up-regulation of MMP1, MMP9, VE-CAD | C | x | (Li et al., 2019h) |
| Glioma | Cell proliferation | miR-4486 sponging  Up-regulation of MDM2, p53 | C | T | (Liu et al., 2019a) |
| Glioma | Cell proliferation and apoptosis | Up-regulation of BCL-2  Down-regulation of BAX, PTEN, PI3K  Inhibition of AKT | C | T | (Guo et al., 2019a) |
| Hepatocellular carcinoma | Cellular proliferation, migration, and invasion | Up-regulation of ZEB1, ZEB2, N-CAD, VIM  Down-regulation of E-CAD | C | x | (Zhang et al., 2016a;Liu et al., 2017b) |
| Hepatocellular carcinoma (From fatty liver disease) | Malignant progression | Activation of STAT6 | C | x | (Wang et al., 2019a) |
| Laryngeal squamous cell carcinoma | Cell proliferation | miR-140 sponging | C | T | (Li et al., 2019k) |
| Lung cancer | Cell proliferation and invasion | Interact with EZH2  Down-regulation of P21 | C | T | (Chen et al., 2017) |
| Lung cancer | Cell proliferation, migration and invasion | miR-154 sponge  Up-regulation of ZEB2 | C | T | (Lingling et al., 2019) |
| Nasopharyngeal carcinoma | Cell migration and invasion | Up-regulation of TGF-B1, MMP2, MMP9 | C | x | (Sun et al., 2018a) |
| Oral cancer | Cell proliferation | Up-regulation of PCNA and Ki67 expression | C | x | (Gao et al., 2019b) |
| Oral cancer | Tumorigenesis | miR-197 sponging  Up-regulation of LIN28, NANOG, OCT4, SOX2 | C | x | (Wu et al., 2019b) |
| Osteosarcoma | Apoptosis | activation of miR-139 sponging  Up-regulation of BCL-2, RUNX  Down-regulation of BAX  Inactivation of CASP3 | C | x | (Wang et al., 2018b) |
| Osteosarcoma | Cell migration and invasion | Up-regulation of ZEB1, ZEB2, VIM  Down-regulation of E-CAD | C | T | (Zhang et al., 2018a) |
| Ovarian cancer | Cell cycle progression and invasion | Up-regulation of CCND1, VIM  Down-regulation of P21, E-CAD | C | T | (Wang et al., 2019c) |
| Ovarian cancer | Cell proliferation and invasion | Up-regulation of β-catenin, CCND1, c-MYC,  Down-regulation of E-CAD  Activation of GSK-3β | C | x | (He et al., 2018) |

An, H.X., Xu, B., Wang, Q., Li, Y.S., Shen, L.F., and Li, S.G. (2018). Up-regulation of long non-coding RNA SNHG6 predicts poor prognosis in renal cell carcinoma. *Eur Rev Med Pharmacol Sci* 22**,** 8624-8629.

Ansari, H., Shahrisa, A., Birgani, Y.T., Birgani, M.T., Hajjari, M., and Asl, J.M. (2019). Long Noncoding RNAs in Colorectal Adenocarcinoma; an in silico Analysis. *Pathol Oncol Res* 25**,** 1387-1394.

Cai, C., Huo, Q., Wang, X., Chen, B., and Yang, Q. (2017). SNHG16 contributes to breast cancer cell migration by competitively binding miR-98 with E2F5. *Biochem Biophys Res Commun* 485**,** 272-278.

Cai, G., Zhu, Q., Yuan, L., and Lan, Q. (2018). LncRNA SNHG6 acts as a prognostic factor to regulate cell proliferation in glioma through targeting p21. *Biomed Pharmacother* 102**,** 452-457.

Cao, X., Xu, J., and Yue, D. (2018). LncRNA-SNHG16 predicts poor prognosis and promotes tumor proliferation through epigenetically silencing p21 in bladder cancer. *Cancer Gene Ther* 25**,** 10-17.

Chang, L., Yuan, Y., Li, C., Guo, T., Qi, H., Xiao, Y., Dong, X., Liu, Z., and Liu, Q. (2016). Upregulation of SNHG6 regulates ZEB1 expression by competitively binding miR-101-3p and interacting with UPF1 in hepatocellular carcinoma. *Cancer Lett* 383**,** 183-194.

Chen, H., Li, M., and Huang, P. (2019a). LncRNA SNHG16 Promotes Hepatocellular Carcinoma Proliferation, Migration and Invasion by Regulating miR-186 Expression. *J Cancer* 10**,** 3571-3581.

Chen, J., Wu, Z., and Zhang, Y. (2019b). LncRNA SNHG3 promotes cell growth by sponging miR-196a-5p and indicates the poor survival in osteosarcoma. *Int J Immunopathol Pharmacol* 33**,** 2058738418820743.

Chen, Q., Zhou, W., Du, S.Q., Gong, D.X., Li, J., Bi, J.B., Li, Z.H., Zhang, Z., Li, Z.L., Liu, X.K., and Kong, C.Z. (2019c). Overexpression of SNHG12 regulates the viability and invasion of renal cell carcinoma cells through modulation of HIF1alpha. *Cancer Cell Int* 19**,** 128.

Chen, S.X., Yin, J.F., Lin, B.C., Su, H.F., Zheng, Z., Xie, C.Y., and Fei, Z.H. (2016). Upregulated expression of long noncoding RNA SNHG15 promotes cell proliferation and invasion through regulates MMP2/MMP9 in patients with GC. *Tumour Biol* 37**,** 6801-6812.

Chen, Y., Lian, Y.J., Ma, Y.Q., Wu, C.J., Zheng, Y.K., and Xie, N.C. (2018). LncRNA SNHG1 promotes alpha-synuclein aggregation and toxicity by targeting miR-15b-5p to activate SIAH1 in human neuroblastoma SH-SY5Y cells. *Neurotoxicology* 68**,** 212-221.

Chen, Y., Peng, Y., Xu, Z., Ge, B., Xiang, X., Zhang, T., Gao, L., Shi, H., Wang, C., and Huang, J. (2019d). Knockdown of lncRNA SNHG7 inhibited cell proliferation and migration in bladder cancer through activating Wnt/beta-catenin pathway. *Pathol Res Pract* 215**,** 302-307.

Chen, Z., Chen, X., Chen, P., Yu, S., Nie, F., Lu, B., Zhang, T., Zhou, Y., Chen, Q., Wei, C., Wang, W., and Wang, Z. (2017). Long non-coding RNA SNHG20 promotes non-small cell lung cancer cell proliferation and migration by epigenetically silencing of P21 expression. *Cell Death Dis* 8**,** e3092.

Cheng, D., Fan, J., Ma, Y., Zhou, Y., Qin, K., Shi, M., and Yang, J. (2019). LncRNA SNHG7 promotes pancreatic cancer proliferation through ID4 by sponging miR-342-3p. *Cell Biosci* 9**,** 28.

Cheng, G., Song, Z., Liu, Y., Xiao, H., Ruan, H., Cao, Q., Wang, K., Xiao, W., Xiong, Z., Liu, D., Chen, K., and Zhang, X. (2020). Long noncoding RNA SNHG12 indicates the prognosis of prostate cancer and accelerates tumorigenesis via sponging miR-133b. *J Cell Physiol* 235**,** 1235-1246.

Chi, J.R., Yu, Z.H., Liu, B.W., Zhang, D., Ge, J., Yu, Y., and Cao, X.C. (2019a). SNHG5 Promotes Breast Cancer Proliferation by Sponging the miR-154-5p/PCNA Axis. *Mol Ther Nucleic Acids* 17**,** 138-149.

Chi, R., Chen, X., Liu, M., Zhang, H., Li, F., Fan, X., Wang, W., and Lu, H. (2019b). Role of SNHG7-miR-653-5p-STAT2 feedback loop in regulating neuroblastoma progression. *J Cell Physiol* 234**,** 13403-13412.

Cui, H.X., Zhang, M.Y., Liu, K., Liu, J., Zhang, Z.L., and Fu, L. (2018). LncRNA SNHG15 promotes proliferation and migration of lung cancer via targeting microRNA-211-3p. *Eur Rev Med Pharmacol Sci* 22**,** 6838-6844.

Cui, N., Liu, J., Xia, H., and Xu, D. (2019). LncRNA SNHG20 contributes to cell proliferation and invasion by upregulating ZFX expression sponging miR-495-3p in gastric cancer. *J Cell Biochem* 120**,** 3114-3123.

Cui, Y., Zhang, F., Zhu, C., Geng, L., Tian, T., and Liu, H. (2017). Upregulated lncRNA SNHG1 contributes to progression of non-small cell lung cancer through inhibition of miR-101-3p and activation of Wnt/beta-catenin signaling pathway. *Oncotarget* 8**,** 17785-17794.

Damas, N.D., Marcatti, M., Come, C., Christensen, L.L., Nielsen, M.M., Baumgartner, R., Gylling, H.M., Maglieri, G., Rundsten, C.F., Seemann, S.E., Rapin, N., Thezenas, S., Vang, S., Orntoft, T., Andersen, C.L., Pedersen, J.S., and Lund, A.H. (2016). SNHG5 promotes colorectal cancer cell survival by counteracting STAU1-mediated mRNA destabilization. *Nat Commun* 7**,** 13875.

Deng, Y., Zhao, F., Zhang, Z., Sun, F., and Wang, M. (2018). Long Noncoding RNA SNHG7 Promotes the Tumor Growth and Epithelial-to-Mesenchymal Transition via Regulation of miR-34a Signals in Osteosarcoma. *Cancer Biother Radiopharm* 33**,** 365-372.

Ding, S., Qu, W., Jiao, Y., Zhang, J., Zhang, C., and Dang, S. (2018). LncRNA SNHG12 promotes the proliferation and metastasis of papillary thyroid carcinoma cells through regulating wnt/beta-catenin signaling pathway. *Cancer Biomark* 22**,** 217-226.

Ding, X., Zhang, Y., Yang, H., Mao, W., Chen, B., Yang, S., Ding, X., Zou, D., Mo, W., He, X., and Zhang, X. (2017). Long non-coding RNAs may serve as biomarkers in breast cancer combined with primary lung cancer. *Oncotarget* 8**,** 58210-58221.

Dong, J., Wang, Q., Li, L., and Xiao-Jin, Z. (2018a). Upregulation of Long Non-Coding RNA Small Nucleolar RNA Host Gene 12 Contributes to Cell Growth and Invasion in Cervical Cancer by Acting as a Sponge for MiR-424-5p. *Cell Physiol Biochem* 45**,** 2086-2094.

Dong, Y.Z., Meng, X.M., and Li, G.S. (2018b). Long non-coding RNA SNHG15 indicates poor prognosis of non-small cell lung cancer and promotes cell proliferation and invasion. *Eur Rev Med Pharmacol Sci* 22**,** 2671-2679.

Du, Y., Kong, C., Zhu, Y., Yu, M., Li, Z., Bi, J., Li, Z., Liu, X., Zhang, Z., and Yu, X. (2018). Knockdown of SNHG15 suppresses renal cell carcinoma proliferation and EMT by regulating the NF-kappaB signaling pathway. *Int J Oncol* 53**,** 384-394.

Fan, R.H., Guo, J.N., Yan, W., Huang, M.D., Zhu, C.L., Yin, Y.M., and Chen, X.F. (2018). Small nucleolar host gene 6 promotes esophageal squamous cell carcinoma cell proliferation and inhibits cell apoptosis. *Oncol Lett* 15**,** 6497-6502.

Fei, F., He, Y., He, S., He, Z., Wang, Y., Wu, G., and Li, M. (2018). LncRNA SNHG3 enhances the malignant progress of glioma through silencing KLF2 and p21. *Biosci Rep* 38.

Feng, F., Chen, A., Huang, J., Xia, Q., Chen, Y., and Jin, X. (2018). Long noncoding RNA SNHG16 contributes to the development of bladder cancer via regulating miR-98/STAT3/Wnt/beta-catenin pathway axis. *J Cell Biochem* 119**,** 9408-9418.

Feng, X., Dong, X., Wu, D., Zhao, H., Xu, C., and Li, H. (2019). Long noncoding RNA small nucleolar RNA host gene 12 promotes papillary thyroid carcinoma cell growth and invasion by targeting miR-16-5p. *Histol Histopathol***,** 18155.

Gao, J., Zeng, K., Liu, Y., Gao, L., and Liu, L. (2019a). LncRNA SNHG5 promotes growth and invasion in melanoma by regulating the miR-26a-5p/TRPC3 pathway. *Onco Targets Ther* 12**,** 169-179.

Gao, P., Fan, R., and Ge, T. (2019b). SNHG20 serves as a predictor for prognosis and promotes cell growth in oral squamous cell carcinoma. *Oncol Lett* 17**,** 951-957.

Gao, X.F., He, H.Q., Zhu, X.B., Xie, S.L., and Cao, Y. (2019c). LncRNA SNHG20 promotes tumorigenesis and cancer stemness in glioblastoma via activating PI3K/Akt/mTOR signaling pathway. *Neoplasma* 66**,** 532-542.

Gao, Y.T., and Zhou, Y.C. (2019). Long non-coding RNA (lncRNA) small nucleolar RNA host gene 7 (SNHG7) promotes breast cancer progression by sponging miRNA-381. *Eur Rev Med Pharmacol Sci* 23**,** 6588-6595.

Ge, J., Wu, X.M., Yang, X.T., Gao, J.M., Wang, F., and Ye, K.F. (2018). Role of long non-coding RNA SNHG1 in occurrence and progression of ovarian carcinoma. *Eur Rev Med Pharmacol Sci* 22**,** 329-335.

Guan, Y.X., Zhang, M.Z., Chen, X.Z., Zhang, Q., Liu, S.Z., and Zhang, Y.L. (2018). Lnc RNA SNHG20 participated in proliferation, invasion, and migration of breast cancer cells via miR-495. *J Cell Biochem* 119**,** 7971-7981.

Guo, H., Yang, S., Li, S., Yan, M., Li, L., and Zhang, H. (2018a). LncRNA SNHG20 promotes cell proliferation and invasion via miR-140-5p-ADAM10 axis in cervical cancer. *Biomed Pharmacother* 102**,** 749-757.

Guo, L.P., Zhang, Z.J., Li, R.T., Li, H.Y., and Cui, Y.Q. (2019a). Influences of LncRNA SNHG20 on proliferation and apoptosis of glioma cells through regulating the PTEN/PI3K/AKT signaling pathway. *Eur Rev Med Pharmacol Sci* 23**,** 253-261.

Guo, T., Wang, H., Liu, P., Xiao, Y., Wu, P., Wang, Y., Chen, B., Zhao, Q., Liu, Z., and Liu, Q. (2018b). SNHG6 Acts as a Genome-Wide Hypomethylation Trigger via Coupling of miR-1297-Mediated S-Adenosylmethionine-Dependent Positive Feedback Loops. *Cancer Res* 78**,** 3849-3864.

Guo, X.B., Yin, H.S., and Wang, J.Y. (2018c). Evaluating the diagnostic and prognostic value of long non-coding RNA SNHG15 in pancreatic ductal adenocarcinoma. *Eur Rev Med Pharmacol Sci* 22**,** 5892-5898.

Guo, Z., Zhang, J., Fan, L., Liu, J., Yu, H., Li, X., and Sun, G. (2019b). Long Noncoding RNA (lncRNA) Small Nucleolar RNA Host Gene 16 (SNHG16) Predicts Poor Prognosis and Sorafenib Resistance in Hepatocellular Carcinoma. *Med Sci Monit* 25**,** 2079-2086.

Han, G.H., Lu, K.J., Wang, P., Ye, J., Ye, Y.Y., and Huang, J.X. (2018). LncRNA SNHG16 predicts poor prognosis in ESCC and promotes cell proliferation and invasion by regulating Wnt/beta-catenin signaling pathway. *Eur Rev Med Pharmacol Sci* 22**,** 3795-3803.

Han, W., Du, X., Liu, M., Wang, J., Sun, L., and Li, Y. (2019a). Increased expression of long non-coding RNA SNHG16 correlates with tumor progression and poor prognosis in non-small cell lung cancer. *Int J Biol Macromol* 121**,** 270-278.

Han, Y., Hu, H., and Zhou, J. (2019b). Knockdown of LncRNA SNHG7 inhibited epithelial-mesenchymal transition in prostate cancer though miR-324-3p/WNT2B axis in vitro. *Pathol Res Pract* 215**,** 152537.

He, B., Bai, Y., Kang, W., Zhang, X., and Jiang, X. (2017). LncRNA SNHG5 regulates imatinib resistance in chronic myeloid leukemia via acting as a CeRNA against MiR-205-5p. *Am J Cancer Res* 7**,** 1704-1713.

He, H.T., Xu, M., Kuang, Y., Han, X.Y., Wang, M.Q., and Yang, Q. (2016). Biomarker and competing endogenous RNA potential of tumor-specific long noncoding RNA in chromophobe renal cell carcinoma. *Onco Targets Ther* 9**,** 6399-6406.

He, S., Zhao, Y., Wang, X., Deng, Y., Wan, Z., Yao, S., and Shen, H. (2018). Up-regulation of long non-coding RNA SNHG20 promotes ovarian cancer progression via Wnt/beta-catenin signaling. *Biosci Rep* 38.

Hong, L., Chen, W., Wu, D., and Wang, Y. (2018). Upregulation of SNHG3 expression associated with poor prognosis and enhances malignant progression of ovarian cancer. *Cancer Biomark* 22**,** 367-374.

Hu, X., Hong, Y., and Shang, C. (2019). Knockdown of long non-coding RNA SNHG5 inhibits malignant cellular phenotypes of glioma via Wnt/CTNNB1 signaling pathway. *J Cancer* 10**,** 1333-1340.

Huang, L., Lin, H., Kang, L., Huang, P., Huang, J., Cai, J., Xian, Z., Zhu, P., Huang, M., Wang, L., Xian, C.J., Wang, J., and Dong, J. (2019). Aberrant expression of long noncoding RNA SNHG15 correlates with liver metastasis and poor survival in colorectal cancer. *J Cell Physiol* 234**,** 7032-7039.

Huang, W., Tian, Y., Dong, S., Cha, Y., Li, J., Guo, X., and Yuan, X. (2017). The long non-coding RNA SNHG3 functions as a competing endogenous RNA to promote malignant development of colorectal cancer. *Oncol Rep* 38**,** 1402-1410.

Jafari-Oliayi, A., and Asadi, M.H. (2019). SNHG6 is upregulated in primary breast cancers and promotes cell cycle progression in breast cancer-derived cell lines. *Cell Oncol (Dordr)* 42**,** 211-221.

Jiang, B., Hailong, S., Yuan, J., Zhao, H., Xia, W., Zha, Z., Bin, W., and Liu, Z. (2018a). Identification of oncogenic long noncoding RNA SNHG12 and DUXAP8 in human bladder cancer through a comprehensive profiling analysis. *Biomed Pharmacother* 108**,** 500-507.

Jiang, H., Li, T., Qu, Y., Wang, X., Li, B., Song, J., Sun, X., Tang, Y., Wan, J., Yu, Y., Zhan, J., and Zhang, H. (2018b). Long non-coding RNA SNHG15 interacts with and stabilizes transcription factor Slug and promotes colon cancer progression. *Cancer Lett* 425**,** 78-87.

Jiang, Z., Jiang, C., and Fang, J. (2018c). Up-regulated lnc-SNHG1 contributes to osteosarcoma progression through sequestration of miR-577 and activation of WNT2B/Wnt/beta-catenin pathway. *Biochem Biophys Res Commun* 495**,** 238-245.

Jin, B., Jin, H., Wu, H.B., Xu, J.J., and Li, B. (2018). Long non-coding RNA SNHG15 promotes CDK14 expression via miR-486 to accelerate non-small cell lung cancer cells progression and metastasis. *J Cell Physiol* 233**,** 7164-7172.

Jin, X.J., Chen, X.J., Zhang, Z.F., Hu, W.S., Ou, R.Y., Li, S., Xue, J.S., Chen, L.L., Hu, Y., and Zhu, H. (2019). Long noncoding RNA SNHG12 promotes the progression of cervical cancer via modulating miR-125b/STAT3 axis. *J Cell Physiol* 234**,** 6624-6632.

Ju, C., Zhou, R., Sun, J., Zhang, F., Tang, X., Chen, K.K., Zhao, J., Lan, X., Lin, S., Zhang, Z., and Lv, X.B. (2018). LncRNA SNHG5 promotes the progression of osteosarcoma by sponging the miR-212-3p/SGK3 axis. *Cancer Cell Int* 18**,** 141.

Kong, Q., and Qiu, M. (2018). Long noncoding RNA SNHG15 promotes human breast cancer proliferation, migration and invasion by sponging miR-211-3p. *Biochem Biophys Res Commun* 495**,** 1594-1600.

Lan, X., and Liu, X. (2019). LncRNA SNHG1 functions as a ceRNA to antagonize the effect of miR-145a-5p on the down-regulation of NUAK1 in nasopharyngeal carcinoma cell. *J Cell Mol Med* 23**,** 2351-2361.

Lei, W., Wang, Z.L., Feng, H.J., Lin, X.D., Li, C.Z., and Fan, D. (2018). Long non-coding RNA SNHG12promotes the proliferation and migration of glioma cells by binding to HuR. *Int J Oncol* 53**,** 1374-1384.

Lei, Y., Shi, Y., Duan, J., Liu, Y., Lv, G., Shi, R., Zhang, F., Yang, Q., and Zhao, W. (2019). Identification of alternative splicing and lncRNA genes in pathogenesis of small cell lung cancer based on their RNA sequencing. *Adv Clin Exp Med* 28**,** 1043-1050.

Li, C., Zhou, L., He, J., Fang, X.Q., Zhu, S.W., and Xiong, M.M. (2016). Increased long noncoding RNA SNHG20 predicts poor prognosis in colorectal cancer. *BMC Cancer* 16**,** 655.

Li, J., and Sun, C.K. (2018). Long noncoding RNA SNHG5 is up-regulated and serves as a potential prognostic biomarker in acute myeloid leukemia. *Eur Rev Med Pharmacol Sci* 22**,** 3342-3347.

Li, J., Sun, S., Chen, W., and Yuan, K. (2019a). Small Nucleolar RNA Host Gene 12 (SNHG12) Promotes Proliferation and Invasion of Laryngeal Cancer Cells via Sponging miR-129-5p and Potentiating WW Domain-Containing E3 Ubiquitin Protein Ligase 1 (WWP1) Expression. *Med Sci Monit* 25**,** 5552-5560.

Li, J., Zhang, Z., Xiong, L., Guo, C., Jiang, T., Zeng, L., Li, G., and Wang, J. (2017). SNHG1 lncRNA negatively regulates miR-199a-3p to enhance CDK7 expression and promote cell proliferation in prostate cancer. *Biochem Biophys Res Commun* 487**,** 146-152.

Li, K., Ma, Y.B., Tian, Y.H., Xu, X.L., Gao, Y., He, Y.Q., Pan, W.T., Zhang, J.W., He, C.J., and Wei, L. (2019b). Silencing lncRNA SNHG6 suppresses proliferation and invasion of breast cancer cells through miR-26a/VASP axis. *Pathol Res Pract* 215**,** 152575.

Li, M., Bian, Z., Jin, G., Zhang, J., Yao, S., Feng, Y., Wang, X., Yin, Y., Fei, B., You, Q., and Huang, Z. (2019c). LncRNA-SNHG15 enhances cell proliferation in colorectal cancer by inhibiting miR-338-3p. *Cancer Med* 8**,** 2404-2413.

Li, M., Bian, Z., Yao, S., Zhang, J., Jin, G., Wang, X., Yin, Y., and Huang, Z. (2018a). Up-regulated expression of SNHG6 predicts poor prognosis in colorectal cancer. *Pathol Res Pract* 214**,** 784-789.

Li, M., Zhang, Y.Y., Shang, J., and Xu, Y.D. (2019d). LncRNA SNHG5 promotes cisplatin resistance in gastric cancer via inhibiting cell apoptosis. *Eur Rev Med Pharmacol Sci* 23**,** 4185-4191.

Li, N., Zhan, X., and Zhan, X. (2018b). The lncRNA SNHG3 regulates energy metabolism of ovarian cancer by an analysis of mitochondrial proteomes. *Gynecol Oncol* 150**,** 343-354.

Li, S., Zhang, S., and Chen, J. (2019e). c-Myc induced upregulation of long non-coding RNA SNHG16 enhances progression and carcinogenesis in oral squamous cell carcinoma. *Cancer Gene Ther* 26**,** 400-410.

Li, W., Xu, W., Song, J.S., Wu, T., and Wang, W.X. (2019f). LncRNA SNHG16 promotes cell proliferation through miR-302a-3p/FGF19 axis in hepatocellular carcinoma. *Neoplasma* 66**,** 397-404.

Li, X., Liu, L., Luo, Y., Cui, S., Chen, W., Zeng, A., Shi, Y., and Luo, L. (2019g). Long non-coding RNA SNHG5 promotes glioma progression via miR-205/E2F3 axis. *Biosci Rep* 39.

Li, X., Xue, Y., Liu, X., Zheng, J., Shen, S., Yang, C., Chen, J., Li, Z., Liu, L., Ma, J., Ma, T., and Liu, Y. (2019h). ZRANB2/SNHG20/FOXK1 Axis regulates Vasculogenic mimicry formation in glioma. *J Exp Clin Cancer Res* 38**,** 68.

Li, X.S., Shen, F.Z., Huang, L.Y., Hui, L., Liu, R.H., Ma, Y.J., and Jin, B.Z. (2019i). lncRNA small nucleolar RNA host gene 20 predicts poor prognosis in glioma and promotes cell proliferation by silencing P21. *Onco Targets Ther* 12**,** 805-814.

Li, Y., Guo, D., Zhao, Y., Ren, M., Lu, G., Wang, Y., Zhang, J., Mi, C., He, S., and Lu, X. (2018c). Long non-coding RNA SNHG5 promotes human hepatocellular carcinoma progression by regulating miR-26a-5p/GSK3beta signal pathway. *Cell Death Dis* 9**,** 888.

Li, Y., Li, D., Zhao, M., Huang, S., Zhang, Q., Lin, H., Wang, W., Li, K., Li, Z., Huang, W., Che, Y., and Huang, C. (2018d). Long noncoding RNA SNHG6 regulates p21 expression via activation of the JNK pathway and regulation of EZH2 in gastric cancer cells. *Life Sci* 208**,** 295-304.

Li, Y., Lu, Y., and Chen, Y. (2019j). Long non-coding RNA SNHG16 affects cell proliferation and predicts a poor prognosis in patients with colorectal cancer via sponging miR-200a-3p. *Biosci Rep* 39.

Li, Y., Xu, J., Guo, Y.N., and Yang, B.B. (2019k). LncRNA SNHG20 promotes the development of laryngeal squamous cell carcinoma by regulating miR-140. *Eur Rev Med Pharmacol Sci* 23**,** 3401-3409.

Li, Y., Zeng, C., Hu, J., Pan, Y., Shan, Y., Liu, B., and Jia, L. (2018e). Long non-coding RNA-SNHG7 acts as a target of miR-34a to increase GALNT7 level and regulate PI3K/Akt/mTOR pathway in colorectal cancer progression. *Journal of hematology & oncology* 11**,** 89-89.

Li, Z., Qiu, R., Qiu, X., and Tian, T. (2018f). SNHG6 Promotes Tumor Growth via Repression of P21 in Colorectal Cancer. *Cell Physiol Biochem* 49**,** 463-478.

Lian, D., Amin, B., Du, D., and Yan, W. (2017). Enhanced expression of the long non-coding RNA SNHG16 contributes to gastric cancer progression and metastasis. *Cancer Biomark* 21**,** 151-160.

Liang, R., Xiao, G., Wang, M., Li, X., Li, Y., Hui, Z., Sun, X., Qin, S., Zhang, B., Du, N., Liu, D., and Ren, H. (2018). SNHG6 functions as a competing endogenous RNA to regulate E2F7 expression by sponging miR-26a-5p in lung adenocarcinoma. *Biomed Pharmacother* 107**,** 1434-1446.

Liao, S., Xing, S., and Ma, Y. (2019). LncRNA SNHG16 sponges miR-98-5p to regulate cellular processes in osteosarcoma. *Cancer Chemother Pharmacol* 83**,** 1065-1074.

Lin, Q., Zheng, H., Xu, J., Zhang, F., and Pan, H. (2019). LncRNA SNHG16 aggravates tumorigenesis and development of hepatocellular carcinoma by sponging miR-4500 and targeting STAT3. *J Cell Biochem*.

Lin, S.X., Jiang, H., Xiang, G.Z., Zhang, W.R., Weng, Y.H., Qiu, F.D., Wu, J., and Wang, H.G. (2018). Up-regulation of long non-coding RNA SNHG1 contributes to proliferation and metastasis in laryngeal squamous cell carcinoma. *Eur Rev Med Pharmacol Sci* 22**,** 1333-1341.

Lingling, J., Xiangao, J., Guiqing, H., Jichan, S., Feifei, S., and Haiyan, Z. (2019). SNHG20 knockdown suppresses proliferation, migration and invasion, and promotes apoptosis in non-small cell lung cancer through acting as a miR-154 sponge. *Biomed Pharmacother* 112**,** 108648.

Liu, J., Cheng, L.G., and Li, H.G. (2019a). LncRNA SNHG20 promoted the proliferation of glioma cells via sponging miR-4486 to regulate the MDM2-p53 pathway. *Eur Rev Med Pharmacol Sci* 23**,** 5323-5331.

Liu, J., Liu, L., Wan, J.X., and Song, Y. (2017a). Long noncoding RNA SNHG20 promotes gastric cancer progression by inhibiting p21 expression and regulating the GSK-3beta/ beta-catenin signaling pathway. *Oncotarget* 8**,** 80700-80708.

Liu, J., Lu, C., Xiao, M., Jiang, F., Qu, L., and Ni, R. (2017b). Long non-coding RNA SNHG20 predicts a poor prognosis for HCC and promotes cell invasion by regulating the epithelial-to-mesenchymal transition. *Biomed Pharmacother* 89**,** 857-863.

Liu, K., Hou, Y., Liu, Y., and Zheng, J. (2017c). LncRNA SNHG15 contributes to proliferation, invasion and autophagy in osteosarcoma cells by sponging miR-141. *J Biomed Sci* 24**,** 46.

Liu, L., Ni, J., and He, X. (2018a). Upregulation of the Long Noncoding RNA SNHG3 Promotes Lung Adenocarcinoma Proliferation. *Dis Markers* 2018**,** 5736716.

Liu, S., Zhang, W., Liu, K., and Liu, Y. (2019b). LncRNA SNHG16 promotes tumor growth of pancreatic cancer by targeting miR-218-5p. *Biomed Pharmacother* 114**,** 108862.

Liu, X., Zheng, J., Xue, Y., Qu, C., Chen, J., Wang, Z., Li, Z., Zhang, L., and Liu, Y. (2018b). Inhibition of TDP43-Mediated SNHG12-miR-195-SOX5 Feedback Loop Impeded Malignant Biological Behaviors of Glioma Cells. *Mol Ther Nucleic Acids* 10**,** 142-158.

Liu, Y., Gu, S., Li, H., Wang, J., Wei, C., and Liu, Q. (2019c). SNHG16 promotes osteosarcoma progression and enhances cisplatin resistance by sponging miR-16 to upregulate ATG4B expression. *Biochem Biophys Res Commun* 518**,** 127-133.

Liu, Y., Li, J., Li, F., Li, M., Shao, Y., and Wu, L. (2019d). SNHG15 functions as a tumor suppressor in thyroid cancer. *J Cell Biochem* 120**,** 6120-6126.

Liu, Y., Li, J., Li, M., Li, F., Shao, Y., and Wu, L. (2019e). microRNA-510-5p promotes thyroid cancer cell proliferation, migration, and invasion through suppressing SNHG15. *J Cell Biochem*.

Liu, Y., Zhou, J., Wang, S., Song, Y., Zhou, J., and Ren, F. (2019f). Long non-coding RNA SNHG12 promotes proliferation and invasion of colorectal cancer cells by acting as a molecular sponge of microRNA-16. *Exp Ther Med* 18**,** 1212-1220.

Liu, Z.B., Tang, C., Jin, X., Liu, S.H., and Pi, W. (2018c). Increased expression of lncRNA SNHG12 predicts a poor prognosis of nasopharyngeal carcinoma and regulates cell proliferation and metastasis by modulating Notch signal pathway. *Cancer Biomark* 23**,** 603-613.

Lu, Q., Shan, S., Li, Y., Zhu, D., Jin, W., and Ren, T. (2018a). Long noncoding RNA SNHG1 promotes non-small cell lung cancer progression by up-regulating MTDH via sponging miR-145-5p. *Faseb j* 32**,** 3957-3967.

Lu, Y.F., Cai, X.L., Li, Z.Z., Lv, J., Xiang, Y.A., Chen, J.J., Chen, W.J., Sun, W.Y., Liu, X.M., and Chen, J.B. (2018b). LncRNA SNHG16 Functions as an Oncogene by Sponging MiR-4518 and Up-Regulating PRMT5 Expression in Glioma. *Cell Physiol Biochem* 45**,** 1975-1985.

Luo, X., Song, Y., Tang, L., Sun, D.H., and Ji, D.G. (2018). LncRNA SNHG7 promotes development of breast cancer by regulating microRNA-186. *Eur Rev Med Pharmacol Sci* 22**,** 7788-7797.

Lv, P., Qiu, X., Gu, Y., Yang, X., Xu, X., and Yang, Y. (2019). Long non-coding RNA SNHG6 enhances cell proliferation, migration and invasion by regulating miR-26a-5p/MAPK6 in breast cancer. *Biomed Pharmacother* 110**,** 294-301.

Ma, X.R., Xu, Y.L., Qian, J., and Wang, Y. (2019). Long non-coding RNA SNHG15 accelerates the progression of non-small cell lung cancer by absorbing miR-211-3p. *Eur Rev Med Pharmacol Sci* 23**,** 1536-1544.

Ma, Y., Xue, Y., Liu, X., Qu, C., Cai, H., Wang, P., Li, Z., Li, Z., and Liu, Y. (2017a). SNHG15 affects the growth of glioma microvascular endothelial cells by negatively regulating miR-153. *Oncol Rep* 38**,** 3265-3277.

Ma, Z., Huang, H., Wang, J., Zhou, Y., Pu, F., Zhao, Q., Peng, P., Hui, B., Ji, H., and Wang, K. (2017b). Long non-coding RNA SNHG15 inhibits P15 and KLF2 expression to promote pancreatic cancer proliferation through EZH2-mediated H3K27me3. *Oncotarget* 8**,** 84153-84167.

Ma, Z., Xue, S., Zeng, B., and Qiu, D. (2018). lncRNA SNHG5 is associated with poor prognosis of bladder cancer and promotes bladder cancer cell proliferation through targeting p27. *Oncol Lett* 15**,** 1924-1930.

Meng, Q., Yang, B.Y., Liu, B., Yang, J.X., and Sun, Y. (2018). Long non-coding RNA SNHG6 promotes glioma tumorigenesis by sponging miR-101-3p. *Int J Biol Markers* 33**,** 148-155.

Meng, S., Jian, Z., Yan, X., Li, J., and Zhang, R. (2019). LncRNA SNHG6 inhibits cell proliferation and metastasis by targeting ETS1 via the PI3K/AKT/mTOR pathway in colorectal cancer. *Mol Med Rep* 20**,** 2541-2548.

Pang, W., Zhai, M., Wang, Y., and Li, Z. (2019). Long noncoding RNA SNHG16 silencing inhibits the aggressiveness of gastric cancer via upregulation of microRNA-628-3p and consequent decrease of NRP1. *Cancer Manag Res* 11**,** 7263-7277.

Peng, H., and Li, H. (2019). The encouraging role of long noncoding RNA small nuclear RNA host gene 16 in epithelial-mesenchymal transition of bladder cancer via directly acting on miR-17-5p/metalloproteinases 3 axis. *Mol Carcinog* 58**,** 1465-1480.

Qi, H., Wang, J., Wang, F., and Ma, H. (2017). Long non-coding RNA SNHG1 promotes cell proliferation and tumorigenesis in colorectal cancer via Wnt/beta-catenin signaling. *Pharmazie* 72**,** 395-401.

Qi, H., Wen, B., Wu, Q., Cheng, W., Lou, J., Wei, J., Huang, J., Yao, X., and Weng, G. (2018). Long noncoding RNA SNHG7 accelerates prostate cancer proliferation and cycle progression through cyclin D1 by sponging miR-503. *Biomed Pharmacother* 102**,** 326-332.

Qu, C., Dai, C., Guo, Y., Qin, R., and Liu, J. (2019). Long noncoding RNA SNHG15 serves as an oncogene and predicts poor prognosis in epithelial ovarian cancer. *Onco Targets Ther* 12**,** 101-111.

Ren, J., Yang, Y., Xue, J., Xi, Z., Hu, L., Pan, S.-J., and Sun, Q. (2018). Long noncoding RNA SNHG7 promotes the progression and growth of glioblastoma via inhibition of miR-5095. *Biochemical and biophysical research communications* 496**,** 712-718.

Ruan, J., Zheng, L., Hu, N., Guan, G., Chen, J., Zhou, X., and Li, M. (2018). Long noncoding RNA SNHG6 promotes osteosarcoma cell proliferation through regulating p21 and KLF2. *Arch Biochem Biophys* 646**,** 128-136.

Ruan, W., Wang, P., Feng, S., Xue, Y., and Li, Y. (2016). Long non-coding RNA small nucleolar RNA host gene 12 (SNHG12) promotes cell proliferation and migration by upregulating angiomotin gene expression in human osteosarcoma cells. *Tumour Biol* 37**,** 4065-4073.

Saeinasab, M., Bahrami, A.R., Gonzalez, J., Marchese, F.P., Martinez, D., Mowla, S.J., Matin, M.M., and Huarte, M. (2019). SNHG15 is a bifunctional MYC-regulated noncoding locus encoding a lncRNA that promotes cell proliferation, invasion and drug resistance in colorectal cancer by interacting with AIF. *J Exp Clin Cancer Res* 38**,** 172.

Shan, Y., Ma, J., Pan, Y., Hu, J., Liu, B., and Jia, L. 2018. LncRNA SNHG7 sponges miR-216b to promote proliferation and liver metastasis of colorectal cancer through upregulating GALNT1. *Cell death & disease* [Online], 9. Available: <http://europepmc.org/abstract/MED/29915311>

<http://europepmc.org/articles/PMC6006356?pdf=render>

<http://europepmc.org/articles/PMC6006356>

<https://doi.org/10.1038/s41419-018-0759-7> [Accessed 2018/06//].

She, K., Huang, J., Zhou, H., Huang, T., Chen, G., and He, J. (2016). lncRNA-SNHG7 promotes the proliferation, migration and invasion and inhibits apoptosis of lung cancer cells by enhancing the FAIM2 expression. *Oncol Rep* 36**,** 2673-2680.

She, K., Yan, H., Huang, J., Zhou, H., and He, J. (2018). miR-193b availability is antagonized by LncRNA-SNHG7 for FAIM2-induced tumour progression in non-small cell lung cancer. *Cell Prolif* 51.

Song, J., Wu, X., Ma, R., Miao, L., Xiong, L., and Zhao, W. (2019). Long noncoding RNA SNHG12 promotes cell proliferation and activates Wnt/beta-catenin signaling in prostate cancer through sponging microRNA-195. *J Cell Biochem* 120**,** 13066-13075.

Sterbova, M., Pazourkova, E., Santorova-Pospisilova, S., Zednikova, I., Tesarova, P., and Korabecna, M. (2019). The use of Human Inflammatory Response and Autoimmunity RT2 lncRNA PCR Array for plasma examination in breast cancer patients prior to therapy. *Neoplasma* 66**,** 641-646.

Su, L., Wu, A., Zhang, W., and Kong, X. (2019a). Silencing long non-coding RNA SNHG6 restrains proliferation, migration and invasion of Wilms' tumour cell lines by regulating miR-15a. *Artif Cells Nanomed Biotechnol* 47**,** 2670-2677.

Su, P., Mu, S., and Wang, Z. (2019b). Long Noncoding RNA SNHG16 Promotes Osteosarcoma Cells Migration and Invasion via Sponging miRNA-340. *DNA Cell Biol* 38**,** 170-175.

Sun, B.Z., Ji, D.G., Feng, Z.X., and Wang, Y. (2019a). Long noncoding RNA SNHG7 represses the expression of RBM5 to strengthen metastasis of hepatocellular carcinoma. *Eur Rev Med Pharmacol Sci* 23**,** 5699-5704.

Sun, C., Sun, Y., and Zhang, E. (2018a). Long non-coding RNA SNHG20 promotes nasopharyngeal carcinoma cell migration and invasion by upregulating TGF-beta1. *Exp Ther Med* 16**,** 4967-4974.

Sun, D., and Fan, X.H. (2019). LncRNA SNHG12 accelerates the progression of ovarian cancer via absorbing miRNA-129 to upregulate SOX4. *Eur Rev Med Pharmacol Sci* 23**,** 2345-2352.

Sun, X., Bai, Y., Yang, C., Hu, S., Hou, Z., and Wang, G. (2019b). Long noncoding RNA SNHG15 enhances the development of colorectal carcinoma via functioning as a ceRNA through miR-141/SIRT1/Wnt/beta-catenin axis. *Artif Cells Nanomed Biotechnol* 47**,** 2536-2544.

Sun, X., Huang, T., Liu, Z., Sun, M., and Luo, S. (2019c). LncRNA SNHG7 contributes to tumorigenesis and progression in breast cancer by interacting with miR-34a through EMT initiation and the Notch-1 pathway. *Eur J Pharmacol* 856**,** 172407.

Sun, Y., Liu, J., Chu, L., Yang, W., Liu, H., Li, C., and Yang, J. (2018b). Long noncoding RNA SNHG12 facilitates the tumorigenesis of glioma through miR-101-3p/FOXP1 axis. *Gene* 676**,** 315-321.

Taherian-Esfahani, Z., Taheri, M., Dashti, S., Kholghi-Oskooei, V., Geranpayeh, L., and Ghafouri-Fard, S. (2019). Assessment of the expression pattern of mTOR-associated lncRNAs and their genomic variants in the patients with breast cancer. *J Cell Physiol* 234**,** 22044-22056.

Tian, T., Qiu, R., and Qiu, X. (2018). SNHG1 promotes cell proliferation by acting as a sponge of miR-145 in colorectal cancer. *Oncotarget* 9**,** 2128-2139.

Wang, B., Li, X., Hu, W., Zhou, Y., and Din, Y. (2019a). Silencing of lncRNA SNHG20 delays the progression of nonalcoholic fatty liver disease to hepatocellular carcinoma via regulating liver Kupffer cells polarization. *IUBMB Life* 71**,** 1952-1961.

Wang, C., Tao, W., Ni, S., and Chen, Q. (2019b). Upregulation of lncRNA snoRNA host gene 6 regulates NUAK family SnF1-like kinase-1 expression by competitively binding microRNA-125b and interacting with Snail1/2 in bladder cancer. *J Cell Biochem* 120**,** 357-367.

Wang, D., Dai, J., Hou, S., and Qian, Y. (2019c). LncRNA SNHG20 predicts a poor prognosis and promotes cell progression in epithelial ovarian cancer. *Biosci Rep* 39.

Wang, D., Zheng, J., Liu, X., Xue, Y., Liu, L., Ma, J., He, Q., Li, Z., Cai, H., and Liu, Y. (2019d). Knockdown of USF1 Inhibits the Vasculogenic Mimicry of Glioma Cells via Stimulating SNHG16/miR-212-3p and linc00667/miR-429 Axis. *Mol Ther Nucleic Acids* 14**,** 465-482.

Wang, J., Cao, L., Wu, J., and Wang, Q. (2018a). Long non-coding RNA SNHG1 regulates NOB1 expression by sponging miR-326 and promotes tumorigenesis in osteosarcoma. *Int J Oncol* 52**,** 77-88.

Wang, J.Z., Xu, C.L., Wu, H., and Shen, S.J. (2017a). LncRNA SNHG12 promotes cell growth and inhibits cell apoptosis in colorectal cancer cells. *Braz J Med Biol Res* 50**,** e6079.

Wang, L., Su, K., Wu, H., Li, J., and Song, D. (2019e). LncRNA SNHG3 regulates laryngeal carcinoma proliferation and migration by modulating the miR-384/WEE1 axis. *Life Sci* 232**,** 116597.

Wang, L., Xu, T., Cui, X., Han, M., Zhou, L.H., Wei, Z.X., Xu, Z.J., and Jiang, Y. (2019f). Downregulation of lncRNA SNHG7 inhibits proliferation and invasion of nasopharyngeal carcinoma cells through repressing ROCK1. *Eur Rev Med Pharmacol Sci* 23**,** 6186-6193.

Wang, M.W., Liu, J., Liu, Q., Xu, Q.H., Li, T.F., Jin, S., and Xia, T.S. (2017b). LncRNA SNHG7 promotes the proliferation and inhibits apoptosis of gastric cancer cells by repressing the P15 and P16 expression. *European review for medical and pharmacological sciences* 21**,** 4613-4622.

Wang, O., Yang, F., Liu, Y., Lv, L., Ma, R., Chen, C., Wang, J., Tan, Q., Cheng, Y., Xia, E., Chen, Y., and Zhang, X. (2017c). C-MYC-induced upregulation of lncRNA SNHG12 regulates cell proliferation, apoptosis and migration in triple-negative breast cancer. *Am J Transl Res* 9**,** 533-545.

Wang, P., Chen, D., Ma, H., and Li, Y. (2017d). LncRNA SNHG12 contributes to multidrug resistance through activating the MAPK/Slug pathway by sponging miR-181a in non-small cell lung cancer. *Oncotarget* 8**,** 84086-84101.

Wang, Q., Li, Q., Zhou, P., Deng, D., Xue, L., Shao, N., Peng, Y., and Zhi, F. (2017e). Upregulation of the long non-coding RNA SNHG1 predicts poor prognosis, promotes cell proliferation and invasion, and reduces apoptosis in glioma. *Biomed Pharmacother* 91**,** 906-911.

Wang, W., Luo, P., Guo, W., Shi, Y., Xu, D., Zheng, H., and Jia, L. (2018b). LncRNA SNHG20 knockdown suppresses the osteosarcoma tumorigenesis through the mitochondrial apoptosis pathway by miR-139/RUNX2 axis. *Biochem Biophys Res Commun* 503**,** 1927-1933.

Wang, X., Hu, K., Chao, Y., and Wang, L. (2019g). LncRNA SNHG16 promotes proliferation, migration and invasion of osteosarcoma cells by targeting miR-1301/BCL9 axis. *Biomed Pharmacother* 114**,** 108798.

Wang, X., Kan, J., Han, J., Zhang, W., Bai, L., and Wu, H. (2019h). LncRNA SNHG16 Functions as an Oncogene by Sponging MiR-135a and Promotes JAK2/STAT3 Signal Pathway in Gastric Cancer. *J Cancer* 10**,** 1013-1022.

Wang, X., Lai, Q., He, J., Li, Q., Ding, J., Lan, Z., Gu, C., Yan, Q., Fang, Y., Zhao, X., and Liu, S. (2019i). LncRNA SNHG6 promotes proliferation, invasion and migration in colorectal cancer cells by activating TGF-beta/Smad signaling pathway via targeting UPF1 and inducing EMT via regulation of ZEB1. *Int J Med Sci* 16**,** 51-59.

Wang, X., Qi, G., Zhang, J., Wu, J., Zhou, N., Li, L., and Ma, J. (2017f). Knockdown of Long Noncoding RNA Small Nucleolar RNA Host Gene 12 Inhibits Cell Growth and Induces Apoptosis by Upregulating miR-138 in Nonsmall Cell Lung Cancer. *DNA Cell Biol* 36**,** 892-900.

Wang, Y., Liang, S., Yu, Y., Shi, Y., and Zheng, H. (2019j). Knockdown of SNHG12 suppresses tumor metastasis and epithelial-mesenchymal transition via the Slug/ZEB2 signaling pathway by targeting miR-218 in NSCLC. *Oncol Lett* 17**,** 2356-2364.

Wang, Y.H., Huo, B.L., Li, C., Ma, G., and Cao, W. (2019k). Knockdown of long noncoding RNA SNHG7 inhibits the proliferation and promotes apoptosis of thyroid cancer cells by downregulating BDNF. *Eur Rev Med Pharmacol Sci* 23**,** 4815-4821.

Wang, Z., Pan, L., Yu, H., and Wang, Y. (2018c). The long non-coding RNA SNHG5 regulates gefitinib resistance in lung adenocarcinoma cells by targetting miR-377/CASP1 axis. *Biosci Rep* 38.

Wang, Z., Wang, Z., Liu, J., and Yang, H. (2018d). Long non-coding RNA SNHG5 sponges miR-26a to promote the tumorigenesis of osteosarcoma by targeting ROCK1. *Biomed Pharmacother* 107**,** 598-605.

Wen, Q., Zhao, L., Wang, T., Lv, N., Cheng, X., Zhang, G., and Bai, L. (2019). LncRNA SNHG16 drives proliferation and invasion of papillary thyroid cancer through modulation of miR-497. *Onco Targets Ther* 12**,** 699-708.

Wu, D.M., Wang, S., Wen, X., Han, X.R., Wang, Y.J., Shen, M., Fan, S.H., Zhang, Z.F., Shan, Q., Li, M.Q., Hu, B., Lu, J., Chen, G.Q., and Zheng, Y.L. (2018). LncRNA SNHG15 acts as a ceRNA to regulate YAP1-Hippo signaling pathway by sponging miR-200a-3p in papillary thyroid carcinoma. *Cell Death Dis* 9**,** 947.

Wu, G., Ju, X., Wang, Y., Li, Z., and Gan, X. (2019a). Up-regulation of SNHG6 activates SERPINH1 expression by competitive binding to miR-139-5p to promote hepatocellular carcinoma progression. *Cell Cycle* 18**,** 1849-1867.

Wu, J., Zhao, W., Wang, Z., Xiang, X., Zhang, S., and Liu, L. (2019b). Long non-coding RNA SNHG20 promotes the tumorigenesis of oral squamous cell carcinoma via targeting miR-197/LIN28 axis. *J Cell Mol Med* 23**,** 680-688.

Wu, P., Tang, Y., Fang, X., Xie, C., Zeng, J., Wang, W., and Zhao, S. (2019c). Metformin Suppresses Hypopharyngeal Cancer Growth by Epigenetically Silencing Long Non-coding RNA SNHG7 in FaDu Cells. *Front Pharmacol* 10**,** 143.

Wu, Y., Deng, Y., Guo, Q., Zhu, J., Cao, L., Guo, X., Xu, F., Weng, W., Ju, X., and Wu, X. (2019d). Long non-coding RNA SNHG6 promotes cell proliferation and migration through sponging miR-4465 in ovarian clear cell carcinoma. *J Cell Mol Med* 23**,** 5025-5036.

Xie, X., Xu, X., Sun, C., and Yu, Z. (2019). Long intergenic noncoding RNA SNHG16 interacts with miR-195 to promote proliferation, invasion and tumorigenesis in hepatocellular carcinoma. *Exp Cell Res* 383**,** 111501.

Xin, L., Zhou, L.Q., Liu, L., Yuan, Y.W., Zhang, H.T., and Zeng, F. (2019). METase promotes cell autophagy via promoting SNHG5 and suppressing miR-20a in gastric cancer. *Int J Biol Macromol* 122**,** 1046-1052.

Xu, C., Hu, C., Wang, Y., and Liu, S. (2019a). Long noncoding RNA SNHG16 promotes human retinoblastoma progression via sponging miR-140-5p. *Biomed Pharmacother* 117**,** 109153.

Xu, C., Zhou, J., Wang, Y., Wang, A., Su, L., Liu, S., and Kang, X. (2019b). Inhibition of malignant human bladder cancer phenotypes through the down-regulation of the long non-coding RNA SNHG7. *J Cancer* 10**,** 539-546.

Xu, F., Zha, G., Wu, Y., Cai, W., and Ao, J. (2018a). Overexpressing lncRNA SNHG16 inhibited HCC proliferation and chemoresistance by functionally sponging hsa-miR-93. *Onco Targets Ther* 11**,** 8855-8863.

Xu, L.J., Yu, X.J., Wei, B., Hui, H.X., Sun, Y., Dai, J., and Chen, X.F. (2018b). LncRNA SNHG7 promotes the proliferation of esophageal cancer cells and inhibits its apoptosis. *European review for medical and pharmacological sciences* 22**,** 2653-2661.

Xu, M., Chen, X., Lin, K., Zeng, K., Liu, X., Xu, X., Pan, B., Xu, T., Sun, L., He, B., Pan, Y., Sun, H., and Wang, S. (2019c). lncRNA SNHG6 regulates EZH2 expression by sponging miR-26a/b and miR-214 in colorectal cancer. *J Hematol Oncol* 12**,** 3.

Xue, W., Li, J., Wang, F., Han, P., Liu, Y., and Cui, B. (2017). A long non-coding RNA expression signature to predict survival of patients with colon adenocarcinoma. *Oncotarget* 8**,** 101298-101308.

Yan, K., Tian, J., Shi, W., Xia, H., and Zhu, Y. (2017a). LncRNA SNHG6 is Associated with Poor Prognosis of Gastric Cancer and Promotes Cell Proliferation and EMT through Epigenetically Silencing p27 and Sponging miR-101-3p. *Cell Physiol Biochem* 42**,** 999-1012.

Yan, Y., Chen, Z., Xiao, Y., Wang, X., and Qian, K. (2019). Long non-coding RNA SNHG6 is upregulated in prostate cancer and predicts poor prognosis. *Mol Biol Rep* 46**,** 2771-2778.

Yan, Y., Fan, Q., Wang, L., Zhou, Y., Li, J., and Zhou, K. (2017b). LncRNA Snhg1, a non-degradable sponge for miR-338, promotes expression of proto-oncogene CST3 in primary esophageal cancer cells. *Oncotarget* 8**,** 35750-35760.

Yang, B.F., Cai, W., and Chen, B. (2018a). LncRNA SNHG12 regulated the proliferation of gastric carcinoma cell BGC-823 by targeting microRNA-199a/b-5p. *Eur Rev Med Pharmacol Sci* 22**,** 1297-1306.

Yang, B.Y., Meng, Q., Sun, Y., Gao, L., and Yang, J.X. (2018b). Long non-coding RNA SNHG16 contributes to glioma malignancy by competitively binding miR-20a-5p with E2F1. *J Biol Regul Homeost Agents* 32**,** 251-261.

Yang, T., Jin, X., Lan, J., and Wang, W. (2019). Long non-coding RNA SNHG16 has Tumor suppressing effect in acute lymphoblastic leukemia by inverse interaction on hsa-miR-124-3p. *IUBMB Life* 71**,** 134-142.

Yang, X.S., Wang, G.X., and Luo, L. (2018c). Long non-coding RNA SNHG16 promotes cell growth and metastasis in ovarian cancer. *Eur Rev Med Pharmacol Sci* 22**,** 616-622.

Ye, J., Tan, L., Fu, Y., Xu, H., Wen, L., Deng, Y., and Liu, K. (2019a). LncRNA SNHG15 promotes hepatocellular carcinoma progression by sponging miR-141-3p. *J Cell Biochem* 120**,** 19775-19783.

Ye, J., Zhang, R., Du, X., Chai, W., and Zhou, Q. (2019b). Long noncoding RNA SNHG16 induces sorafenib resistance in hepatocellular carcinoma cells through sponging miR-140-5p. *Onco Targets Ther* 12**,** 415-422.

You, J., Fang, N., Gu, J., Zhang, Y., Li, X., Zu, L., and Zhou, Q. (2014). Noncoding RNA small nucleolar RNA host gene 1 promote cell proliferation in nonsmall cell lung cancer. *Indian J Cancer* 51 Suppl 3**,** e99-e102.

Yu, C., Sun, J., Leng, X., and Yang, J. (2019a). Long noncoding RNA SNHG6 functions as a competing endogenous RNA by sponging miR-181a-5p to regulate E2F5 expression in colorectal cancer. *Cancer Manag Res* 11**,** 611-624.

Yu, J., Shen, J., Qiao, X., Cao, L., Yang, Z., Ye, H., Xi, C., Zhou, Q., Wang, P., and Gong, Z. (2019b). SNHG20/miR-140-5p/NDRG3 axis contributes to 5-fluorouracil resistance in gastric cancer. *Oncol Lett* 18**,** 1337-1343.

Yu, Y., Chen, F., Yang, Y., Jin, Y., Shi, J., Han, S., Chu, P., Lu, J., Tai, J., Wang, S., Yang, W., Wang, H., Guo, Y., and Ni, X. (2019c). lncRNA SNHG16 is associated with proliferation and poor prognosis of pediatric neuroblastoma. *Int J Oncol* 55**,** 93-102.

Zhang, C., Jiang, F., Su, C., Xie, P., and Xu, L. (2019a). Upregulation of long noncoding RNA SNHG20 promotes cell growth and metastasis in esophageal squamous cell carcinoma via modulating ATM-JAK-PD-L1 pathway. *J Cell Biochem*.

Zhang, C., Zhu, B., Li, X.B., Cao, Y.Q., Yang, J.C., Li, X., Liu, Y.X., and Wang, Y.B. (2019b). Long non-coding RNA SNHG7 promotes migration and invasion of melanoma via upregulating SOX4. *Eur Rev Med Pharmacol Sci* 23**,** 4828-4834.

Zhang, D., Cao, C., Liu, L., and Wu, D. (2016a). Up-regulation of LncRNA SNHG20 Predicts Poor Prognosis in Hepatocellular Carcinoma. *J Cancer* 7**,** 608-617.

Zhang, G.D., Gai, P.Z., Liao, G.Y., and Li, Y. (2019c). LncRNA SNHG7 participates in osteosarcoma progression by down-regulating p53 via binding to DNMT1. *Eur Rev Med Pharmacol Sci* 23**,** 3602-3610.

Zhang, H., and Lu, W. (2018). LncRNA SNHG12 regulates gastric cancer progression by acting as a molecular sponge of miR320. *Mol Med Rep* 17**,** 2743-2749.

Zhang, H., Zhou, D., Ying, M., Chen, M., Chen, P., Chen, Z., and Zhang, F. (2016b). Expression of Long Non-Coding RNA (lncRNA) Small Nucleolar RNA Host Gene 1 (SNHG1) Exacerbates Hepatocellular Carcinoma Through Suppressing miR-195. *Med Sci Monit* 22**,** 4820-4829.

Zhang, J., Ju, C., Zhang, W., and Xie, L. (2018a). LncRNA SNHG20 is associated with clinical progression and enhances cell migration and invasion in osteosarcoma. *IUBMB Life* 70**,** 1115-1121.

Zhang, J.H., Wei, H.W., and Yang, H.G. (2016c). Long noncoding RNA SNHG15, a potential prognostic biomarker for hepatocellular carcinoma. *Eur Rev Med Pharmacol Sci* 20**,** 1720-1724.

Zhang, K., Chen, J., Song, H., and Chen, L.B. (2018b). SNHG16/miR-140-5p axis promotes esophagus cancer cell proliferation, migration and EMT formation through regulating ZEB1. *Oncotarget* 9**,** 1028-1040.

Zhang, M., Duan, W., and Sun, W. (2019d). LncRNA SNHG6 promotes the migration, invasion, and epithelial-mesenchymal transition of colorectal cancer cells by miR-26a/EZH2 axis. *Onco Targets Ther* 12**,** 3349-3360.

Zhang, M., Li, Y., Wang, H., Yu, W., Lin, S., and Guo, J. (2019e). LncRNA SNHG5 affects cell proliferation, metastasis and migration of colorectal cancer through regulating miR-132-3p/CREB5. *Cancer Biol Ther* 20**,** 524-536.

Zhang, M., Wang, W., Li, T., Yu, X., Zhu, Y., Ding, F., Li, D., and Yang, T. (2016d). Long noncoding RNA SNHG1 predicts a poor prognosis and promotes hepatocellular carcinoma tumorigenesis. *Biomed Pharmacother* 80**,** 73-79.

Zhang, P.F., Wang, F., Wu, J., Wu, Y., Huang, W., Liu, D., Huang, X.Y., Zhang, X.M., and Ke, A.W. (2019f). LncRNA SNHG3 induces EMT and sorafenib resistance by modulating the miR-128/CD151 pathway in hepatocellular carcinoma. *J Cell Physiol* 234**,** 2788-2794.

Zhang, T., Cao, C., Wu, D., and Liu, L. (2016e). SNHG3 correlates with malignant status and poor prognosis in hepatocellular carcinoma. *Tumour Biol* 37**,** 2379-2385.

Zhang, Y., Jin, X., Wang, Z., Zhang, X., Liu, S., and Liu, G. (2017). Downregulation of SNHG1 suppresses cell proliferation and invasion by regulating Notch signaling pathway in esophageal squamous cell cancer. *Cancer Biomark* 21**,** 89-96.

Zhang, Y., Li, R., Ding, X., Zhang, K., and Qin, W. (2019g). Upregulation of long non-coding RNA SNHG6 promote esophageal squamous cell carcinoma cell malignancy and its diagnostic value. *Am J Transl Res* 11**,** 1084-1091.

Zhang, Y., Zhang, D., Lv, J., Wang, S., and Zhang, Q. (2019h). LncRNA SNHG15 acts as an oncogene in prostate cancer by regulating miR-338-3p/FKBP1A axis. *Gene* 705**,** 44-50.

Zhao, G., Wang, S., Liang, X., Wang, C., and Peng, B. (2019). Oncogenic role of long non-coding RNA SNHG12 in gastric cancer cells by targeting miR-16. *Exp Ther Med* 18**,** 199-208.

Zhao, L., Han, T., Li, Y., Sun, J., Zhang, S., Liu, Y., Shan, B., Zheng, D., and Shi, J. (2017). The lncRNA SNHG5/miR-32 axis regulates gastric cancer cell proliferation and migration by targeting KLF4. *Faseb j* 31**,** 893-903.

Zhao, Q., and Fan, C. (2019). A novel risk score system for assessment of ovarian cancer based on co-expression network analysis and expression level of five lncRNAs. *BMC Med Genet* 20**,** 103.

Zhao, Q., Gao, S., Du, Q., and Liu, Y. (2018a). Long non-coding RNA SNHG20 promotes bladder cancer via activating the Wnt/beta-catenin signalling pathway. *Int J Mol Med* 42**,** 2839-2848.

Zhao, W., Fu, H., Zhang, S., Sun, S., and Liu, Y. (2018b). LncRNA SNHG16 drives proliferation, migration, and invasion of hemangioma endothelial cell through modulation of miR-520d-3p/STAT3 axis. *Cancer Med*.

Zhao, Y., Qin, Z.S., Feng, Y., Tang, X.J., Zhang, T., and Yang, L. (2018c). Long non-coding RNA (lncRNA) small nucleolar RNA host gene 1 (SNHG1) promote cell proliferation in colorectal cancer by affecting P53. *Eur Rev Med Pharmacol Sci* 22**,** 976-984.

Zheng, S., Jiang, F., Ge, D., Tang, J., Chen, H., Yang, J., Yao, Y., Yan, J., Qiu, J., Yin, Z., Ni, Y., Zhao, L., Chen, X., Li, H., and Yang, L. (2019). LncRNA SNHG3/miRNA-151a-3p/RAB22A axis regulates invasion and migration of osteosarcoma. *Biomed Pharmacother* 112**,** 108695.

Zhong, J.H., Xiang, X., Wang, Y.Y., Liu, X., Qi, L.N., Luo, C.P., Wei, W.E., You, X.M., Ma, L., Xiang, B.D., and Li, L.Q. (2020). The lncRNA SNHG16 affects prognosis in hepatocellular carcinoma by regulating p62 expression. *J Cell Physiol* 235**,** 1090-1102.

Zhong, X., Long, Z., Wu, S., Xiao, M., and Hu, W. (2018). LncRNA-SNHG7 regulates proliferation, apoptosis and invasion of bladder cancer cells assurance guidelines. *J buon* 23**,** 776-781.

Zhou, B., Li, L., Li, Y., Sun, H., and Zeng, C. (2018a). Long noncoding RNA SNHG12 mediates doxorubicin resistance of osteosarcoma via miR-320a/MCL1 axis. *Biomed Pharmacother* 106**,** 850-857.

Zhou, S., Yu, L., Xiong, M., and Dai, G. (2018b). LncRNA SNHG12 promotes tumorigenesis and metastasis in osteosarcoma by upregulating Notch2 by sponging miR-195-5p. *Biochem Biophys Res Commun* 495**,** 1822-1832.

Zhou, X.Y., Liu, H., Ding, Z.B., Xi, H.P., and Wang, G.W. (2019a). lncRNA SNHG16 Exerts Oncogenic Functions in Promoting Proliferation of Glioma Through Suppressing p21. *Pathol Oncol Res*.

Zhou, X.Y., Liu, H., Ding, Z.B., Xi, H.P., and Wang, G.W. (2019b). lncRNA SNHG16 promotes glioma tumorigenicity through miR-373/EGFR axis by activating PI3K/AKT pathway. *Genomics*.

Zhu, C., Cheng, D., Qiu, X., Zhuang, M., and Liu, Z. (2018a). Long Noncoding RNA SNHG16 Promotes Cell Proliferation by Sponging MicroRNA-205 and Upregulating ZEB1 Expression in Osteosarcoma. *Cell Physiol Biochem* 51**,** 429-440.

Zhu, H., Zeng, Y., Zhou, C.C., and Ye, W. (2018b). SNHG16/miR-216-5p/ZEB1 signal pathway contributes to the tumorigenesis of cervical cancer cells. *Arch Biochem Biophys* 637**,** 1-8.

Zhu, L., Zhang, X., Fu, X., Li, Z., Sun, Z., Wu, J., Wang, X., Wang, F., Li, X., Niu, S., Ding, M., Yang, Z., Yang, W., Yin, M., Zhang, L., and Zhang, M. (2019a). c-Myc mediated upregulation of long noncoding RNA SNHG12 regulates proliferation and drug sensitivity in natural killer/T-cell lymphoma. *J Cell Biochem* 120**,** 12628-12637.

Zhu, X., Yang, G., Xu, J., and Zhang, C. (2019b). Silencing of SNHG6 induced cell autophagy by targeting miR-26a-5p/ULK1 signaling pathway in human osteosarcoma. *Cancer Cell Int* 19**,** 82.

Zhu, Y., Xing, Y., Chi, F., Sun, W., Zhang, Z., and Piao, D. (2018c). Long noncoding RNA SNHG6 promotes the progression of colorectal cancer through sponging miR-760 and activation of FOXC1. *Onco Targets Ther* 11**,** 5743-5752.
